# SRSF3 shapes the structure of miR‐17‐92 cluster RNA and promotes selective processing of miR‐17 and miR‐20a

**DOI:** 10.15252/embr.202256021

**Published:** 2023-06-12

**Authors:** Madara Ratnadiwakara, Mohamed NM Bahrudeen, Erika Aikio, Piia Takabe, Rebekah M Engel, Zileena Zahir, Thierry Jardé, Paul J McMurrick, Helen E Abud, Minna‐Liisa Änkö

**Affiliations:** ^1^ Hudson Institute of Medical Research Clayton Vic. Australia; ^2^ Department of Molecular and Translational Science, School of Clinical Sciences Monash University Clayton Vic. Australia; ^3^ Faculty of Medicine and Health Technology Tampere University Tampere Finland; ^4^ Department of Anatomy and Developmental Biology Monash University Clayton Vic. Australia; ^5^ Development and Stem Cells Program Monash Biomedicine Discovery Institute Clayton Vic. Australia; ^6^ Department of Surgery, Cabrini Health Cabrini Monash University Malvern Vic. Australia

**Keywords:** colorectal cancer, miR‐17‐92, pri‐miRNA processing, serine‐arginine rich splicing factor 3, SHAPE‐MaP, Cancer, RNA Biology

## Abstract

MicroRNA (miRNA) biogenesis is tightly regulated to maintain distinct miRNA expression patterns. Almost half of mammalian miRNAs are generated from miRNA clusters, but this process is not well understood. We show here that Serine‐arginine rich splicing factor 3 (SRSF3) controls the processing of miR‐17‐92 cluster miRNAs in pluripotent and cancer cells. SRSF3 binding to multiple CNNC motifs downstream of Drosha cleavage sites within miR‐17‐92 is required for the efficient processing of the cluster. SRSF3 depletion specifically compromises the processing of two paralog miRNAs, miR‐17 and miR‐20a. In addition to SRSF3 binding to the CNNC sites, the SRSF3 RS‐domain is essential for miR‐17‐92 processing. SHAPE‐MaP probing demonstrates that SRSF3 binding disrupts local and distant base pairing, resulting in global changes in miR‐17‐92 RNA structure. Our data suggest a model where SRSF3 binding, and potentially its RS‐domain interactions, may facilitate an RNA structure that promotes miR‐17‐92 processing. SRSF3‐mediated increase in miR‐17/20a levels inhibits the cell cycle inhibitor p21, promoting self‐renewal in normal and cancer cells. The SRSF3‐miR‐17‐92‐p21 pathway operates in colorectal cancer, linking SRSF3‐mediated pri‐miRNA processing and cancer pathogenesis.

## Graphical abstract

SRSF3 binds to CNNC motifs within the miR‐17‐92 cluster and promotes the processing of specific cluster miRNAs. SRSF3 binding changes miR‐17‐92 structure, and this may impact miR‐17‐92 processing.

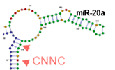

## Introduction

MicroRNA (miRNA) biogenesis is a tightly regulated multistep process involving two consecutive cleavage steps of the miRNA stem‐loop that ultimately determine the amount of mature miRNA in the cell (Siomi & Siomi, 2010). First, the RNase III enzyme Drosha cleaves the pri‐miRNA in the nucleus to generate a pre‐miRNA followed by a Dicer cleavage in the cytoplasm to give rise to a miRNA duplex (Bartel, 2018). Specific sequence motifs within the pri‐miRNA have been shown to affect the pri‐miRNA cleavage efficiency, including an apical GUG/UGU, a GC and UG motifs 13 and 14 nt upstream of the Drosha cleavage site, respectively, and a CNNC motif 16–24 nt downstream of the Drosha cleavage site (Auyeung *et al*, 2013; Conrad *et al*, 2014; Louloupi *et al*, 2017; Roden *et al*, 2017; Kim *et al*, 2018). The CNNC motif was previously identified as the consensus binding motif for Serine‐arginine rich splicing factor 3 (SRSF3), an RNA binding protein (RBP) with multiple functions in mRNA metabolism (Änkö *et al*, 2012; Ratnadiwakara *et al*, 2018). In subsequent studies, binding of SRSF3 to the CNNC motif 17–18 nt downstream of the Drosha cleavage site was shown to enhance the processing of pri‐miR‐30a/c and miR‐16 (Auyeung *et al*, 2013; Fernandez *et al*, 2017; Kim *et al*, 2018). Mechanistically, SRSF3 has been shown to promote the productive processing of pri‐miRNAs *in vitro* by suppressing unproductive 5′ nick and reverse processing (Kim *et al*, 2021). As 60% of the human miRNAs conserved to mouse contain a CNNC motif within the 17–18 nt window (Auyeung *et al*, 2013), and SRSF3 enhances the Drosha cleavage efficiency of hundreds of miRNAs *in vitro* (Kim *et al*, 2021), SRSF3 may play a broad role in modulating efficiency of pri‐miRNA processing during development and in adult tissues.

Polycistronic miRNAs account for ~ 50% of all miRNAs in mammals (Kim *et al*, 2009). The polycistronic clusters often contain paralog miRNAs of the same miRNA family targeting similar sets of mRNAs and allowing efficient gate‐keeping of cellular processes through miRNA regulation (Wang *et al*, 2008; Khuu *et al*, 2016). Several miRNA clusters have been found to be essential for normal development and play a role in disease pathology (Khuu *et al*, 2016). Although polycistronic miRNAs are critical in human development, homeostasis and disease, we know surprisingly little of how their processing is regulated post‐transcriptionally. Importantly, miRNAs produced from a common primary transcript are present at highly varying levels in cells and tissues, suggesting that the processing of individual miRNAs within clusters is differentially regulated in the cellular context (Guil & Cáceres, 2007; Truscott *et al*, 2016; Louloupi *et al*, 2017). For example, hnRNPA1 was shown to specifically bind to the conserved terminal loop of miR‐18a within miR‐17‐92 cluster and promote its processing independent of other miRNAs of the cluster (Guil & Cáceres, 2007).

The miR‐17‐92 cluster comprises six miRNA stem‐loops giving rise to miR‐17, miR‐18a, miR‐19a, miR‐20a, miR‐19b‐1 and miR‐92a‐1. The mature miRNA sequences and their organisation within the miR‐17‐92 cluster are fully conserved in vertebrates (Mendell, 2008). The miRNAs produced from miR‐17‐92 and its paralog clusters miR‐106a‐363 and miR‐106b‐25 are classified into four miRNA families based on their seed sequences: the miR‐17, miR‐18, miR‐19 and miR‐92 families. Genetic studies in mice have shown that the loss of miR‐17‐92 leads to perinatal death, while the ablation of miR‐106a‐363 and/or miR‐106b‐25 results in no obvious phenotype (Ventura *et al*, 2008). This suggests that while there is a functional cooperation between the three clusters, miR‐17‐92 may control pathways that are critical for cell survival. Accordingly, the miR‐17‐92 cluster is fundamental for embryonic stem (ES) cell self‐renewal and proliferation (Gunaratne, 2009). It is also one of the first identified oncogenic miRNAs, hence called OncomiR‐1 (He *et al*, 2005). Dysregulation of miR‐17‐92 miRNAs has been reported in various cancers, including colorectal cancer (CRC; Hayashita *et al*, 2005; Mendell, 2008; Yu *et al*, 2012). Similar to their functions in ES cells, the miR‐17‐92 cluster miRNAs enhance cell proliferation and cell cycle progression in tumour cells (Ranji *et al*, 2013; Zhang *et al*, 2018). In CRC cells, miR‐17/20a upregulation promotes transformation, tumour progression, cell invasion and migration, and a high level of miR‐17/20a is associated with metastatic CRC (Luo *et al*, 2012; Cheng *et al*, 2016; Huang *et al*, 2019; Xu *et al*, 2019b).

Although SRSF3 is a well‐established modulator of pri‐miRNA processing, the functional consequences of SRSF3‐mediated enhanced miRNA processing *in vivo* are poorly understood. Furthermore, the role of SRSF3 in the processing of miRNA clusters has not been investigated. We have previously demonstrated that SRSF3 is essential for self‐renewal in mouse pluripotent stem cells (Ratnadiwakara *et al*, 2018). SRSF3 enhances cell proliferation during somatic reprogramming and in pluripotent cells similar to that observed in various human cancer cell lines (Jia *et al*, 2010; Ajiro *et al*, 2015; Ratnadiwakara *et al*, 2018). Here, we demonstrate that SRSF3 binding to multiple positions within miR‐17‐92 cluster is required for the efficient processing of the cluster, most notably miR‐17 and miR‐20a. SRSF3‐mediated enhancement in miR‐17 and miR‐20a processing promoted self‐renewal, distinguishing poorly differentiated stem cell‐like colorectal tumours. RNA structure probing by SHAPE‐MaP (selective 2′‐hydroxyl acylation analysed by primer extension and mutational profiling) uncovered how SRSF3 rearranged the miR‐17‐92 RNA structure through binding to multiple sites along the pri‐miRNA. The effects of SRSF3 depended on both the CNNC sites and RS‐domain suggesting a model where SRSF3 binding, and potentially its RS‐domain interactions, may facilitate an RNA structure that alters miR‐17‐92 processing. The enhancement of miR‐17/20 processing following SRSF3 overexpression led to altered expression of their target mRNAs encoding key cell cycle regulators in mouse pluripotent cells, human cancer cell lines and primary colorectal tumours. These data mechanistically and functionally link SRSF3‐mediated pri‐miRNA processing to hallmark features of cancer.

## Results

### SRSF3 regulates differential processing of polycistronic miRNAs in pluripotent stem cells

**Figure 1:**
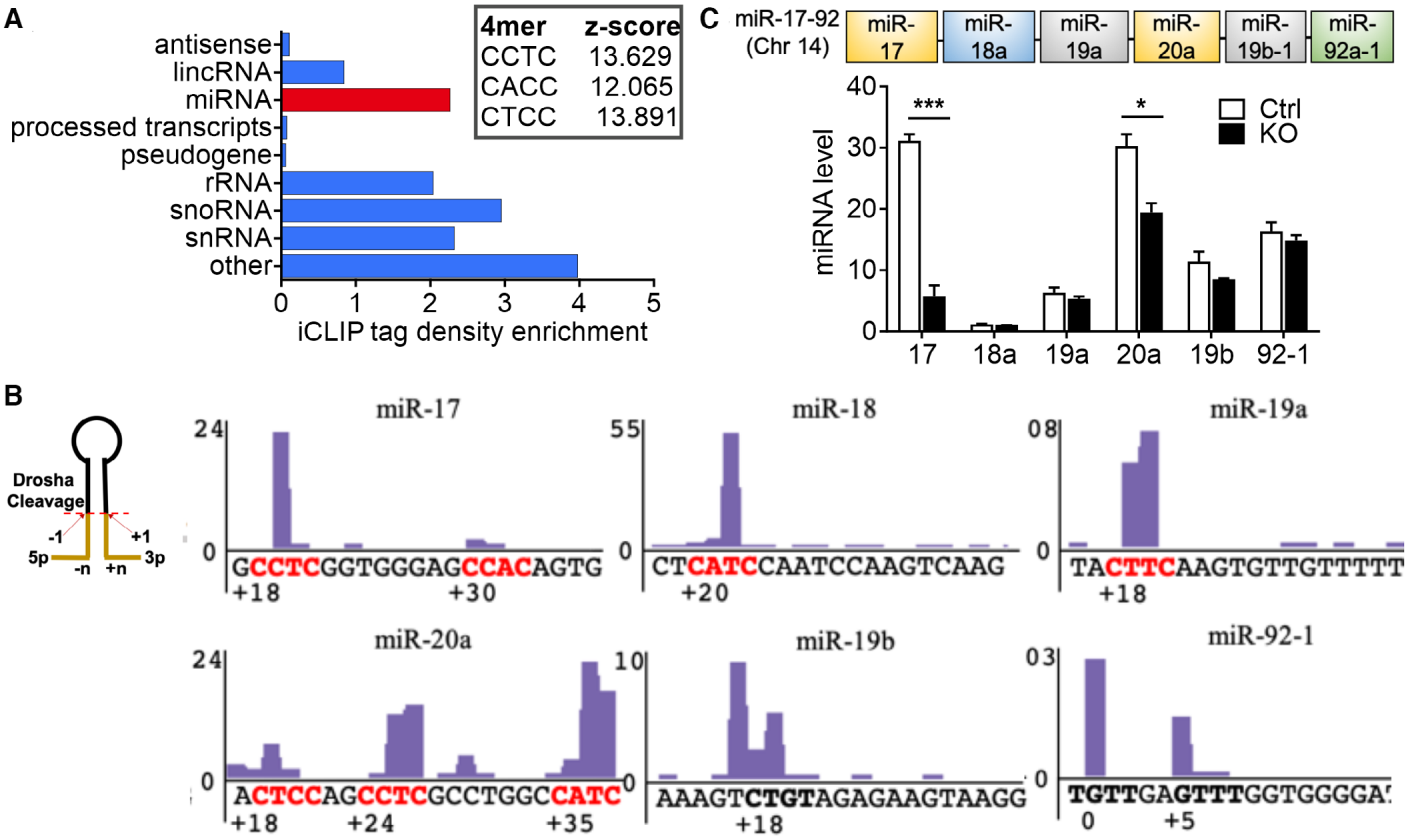
SRSF3 binds to miR‐17‐92 and affects the mature miRNA levels in pluripotent stem cells ADistribution of significant SRSF3 crosslink sites (FDR < 0.05) across noncoding transcript types. *Inset*: top three enriched tetramers around SRSF3 crosslink sites within miRNA transcripts. iCLIP data from (Ratnadiwakara *et al*, 2018).BSRSF3 iCLIP binding peaks within the miR‐17‐92 cluster in mouse ES cells. The 3p position is counted from the 3′ Drosha cleavage site as described in (Roden *et al*, 2017). The CNNC motifs are marked in red. iCLIP data from (Ratnadiwakara *et al*, 2018).C
*Top*: Schematic illustration of the miR‐17‐92 cluster with colours marking miRNAs belonging to the same miRNA family. *Bottom*: TaqMan analysis of the mature miR‐17‐92 miRNAs in *Srsf3* knockout (KO) and control (Ctrl) iPSCs (*P < 0.05, ***P < 0.001, two‐tailed unpaired Student's *t*‐test, data as mean ± SEM, *n* = 3, biological replicates).

**Figure EV1:**
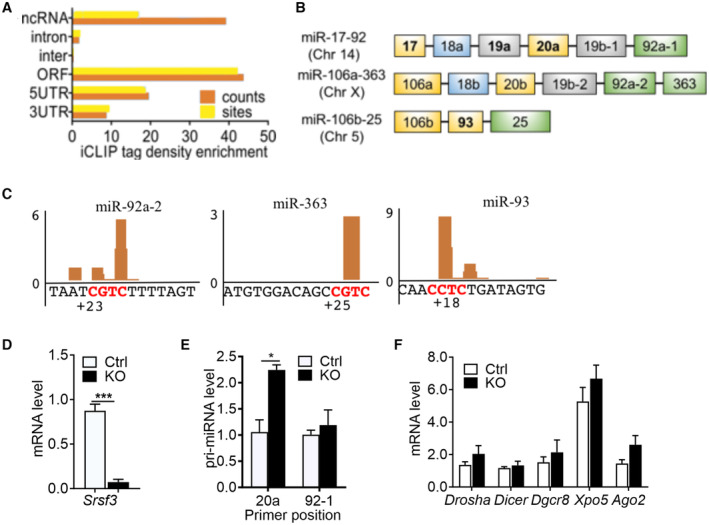
SRSF3 binds to miR‐17‐92 miRNA and its paralog clusters and affects the mature miRNA levels in pluripotent stem cells ADistribution of significant SRSF3 crosslink sites (FDR < 0.05) over different transcript regions normalised to feature length. Data from (Ratnadiwakara *et al*, 2018).BSchematic illustration of three paralog clusters miR‐17‐92, miR‐106a‐363 and miR‐106b‐25. The colours mark miRNAs of the same family. MicroRNAs bound by SRSF3 at 16–18 nt downstream of the Drosha cleavage site based on data in Ratnadiwakara *et al* (2018) are marked in bold.CSRSF3 iCLIP binding peaks within the components of paralogue clusters miR‐106a‐363 and miR‐106b‐25 in mouse ES cells. The CNNC motifs are marked in red colour. The 3p position is counted from the 3′ Drosha cleavage site as described in (Auyeung *et al*, 2013), iCLIP data from (Ratnadiwakara *et al*, 2018).DRT–qPCR quantification of *Srsf3* expression in *Srsf3*‐knockout (KO) and control (Ctrl) iPSCs (***P < 0.001, two‐tailed unpaired Student's *t*‐test, data as mean ± SEM, *n* = 3, biological replicates).ERT–qPCR quantification of pri‐miRNA expression in *Srsf3*‐KO (KO) and control (Ctrl) iPSCs. Two different primer pairs around miR‐20a and miR‐92‐1 stem‐loop regions were used (*P < 0.05, two‐tailed unpaired Student's *t*‐test, data as mean ± SEM, *n* = 3, biological replicates).FRT–qPCR quantification of *Drosha*, *Dicer*, *Dgcr8*, *Xpo5* and *Ago2* mRNA levels in *Srsf3*‐KO (KO) and control (Ctrl) iPSCs (*P* > 0.05 for all genes analysed, two‐tailed unpaired Student's *t*‐test, data as mean ± SEM, *n* = 3, biological replicates).

We previously generated a reprogrammable tamoxifen‐inducible *Srsf3* knockout mouse model and defined an SRSF3‐regulated network of coding mRNAs with key roles in pluripotency (Ratnadiwakara *et al*, 2018). In addition to protein‐coding mRNAs, abundant SRSF3 binding sites were detected in noncoding RNAs (ncRNAs) in both mouse embryonic stem (ES) and P19 teratocarcinoma cells (Figs 1A and EV1A; Änkö *et al*, 2012; Müller‐McNicoll *et al*, 2016), but the functional relevance of SRSF3 binding to ncRNAs in self‐renewing cells has not been investigated. The consensus tetramer binding motif within ncRNAs in pluripotent cells was consistent with the previously identified CNNC motif downstream of pri‐miRNA stem‐loops (Fig 1A; Auyeung *et al*, 2013). Accordingly, miRNAs were among the most enriched ncRNA classes bound by SRSF3 with abundant binding to polycistronic miRNA clusters including the well‐studied miR‐17‐92 that controls proliferation and self‐renewal of ES cells (Gunaratne, 2009). The overlapping roles of miR‐17‐92 and SRSF3 in promoting self‐renewal (Jia *et al*, 2010; Ratnadiwakara *et al*, 2018) prompted us to further investigate the role of SRSF3 in processing of the polycistronic miR‐17‐92.

Previously, SRSF3 was shown to bind to CNNC motifs located in pri‐miRNAs 16‐24 nt downstream of 3′ Drosha cleavage sites and enhance pri‐miRNA processing (Auyeung *et al*, 2013; Kim *et al*, 2018). However, the role of SRSF3 in the processing of miRNA clusters has not been investigated. In mouse pluripotent stem cells, SRSF3 binding sites within the miR‐17‐92 cluster mapped downstream of miR‐17 (at +18 from Drosha cleavage site), miR‐18a (+20), miR‐19a (+18) and miR‐20a (+18), each overlapping a CNNC motif (Fig 1B). An SRSF3 iCLIP binding peak but no CNNC consensus sequence was also detected downstream of miR‐19b (+18). Within miR‐17a and miR‐20a, additional CNNC containing SRSF3 binding peaks were detected outside the optimal 17–18 nt window downstream of Drosha cleavage site (miR‐17 +30, miR‐20a +24 and +35) (Fig 1B). SRSF3 binding peaks were detected also within the two miR‐17‐92 paralog clusters, miR‐106a‐363 and miR‐106b‐25, in miR‐92a‐2 (CNNC at +23), miR‐363 (+25) and miR‐93 (+18) (Fig EV1B–C). Of these, miR‐93 belongs to the miR‐17 family together with miR‐17 and miR‐20a.

To assess whether SRSF3 binding within the primary miR‐17‐92 transcript affects mature miRNA levels, we measured the abundance of pri‐ and mature miRNAs following SRSF3 depletion. SRSF3 depletion did not alter miR‐17‐92 transcript abundance or the expression of miRNA biogenesis pathway components *Drosha*, *Dicer*, *Dgcr8*, *Xpo5* and *Ago2* (Fig EV1D–F). The steady‐state levels of mature miRNAs produced from the miR‐17‐92 transcript differed by up to 30‐fold in the control cells (miR‐18a vs miR‐17/20a), suggesting post‐transcriptional regulation of the individual miRNAs (Fig 1C, white bars). SRSF3 depletion led to a significant decrease in the levels of miR‐17 and miR‐20a with no significant effect on the abundance of the other miRNAs produced from the cluster (Fig 1C). Thus, SRSF3 binding downstream of Drosha cleavage sites within the miR‐17‐92 RNA preferentially promoted the processing of miR‐17 and miR‐20a.

### SRSF3 binding to the CNNC sites and SRSF3 RS‐domain are essential for the efficient processing of miR‐17‐92

**Figure 2:**
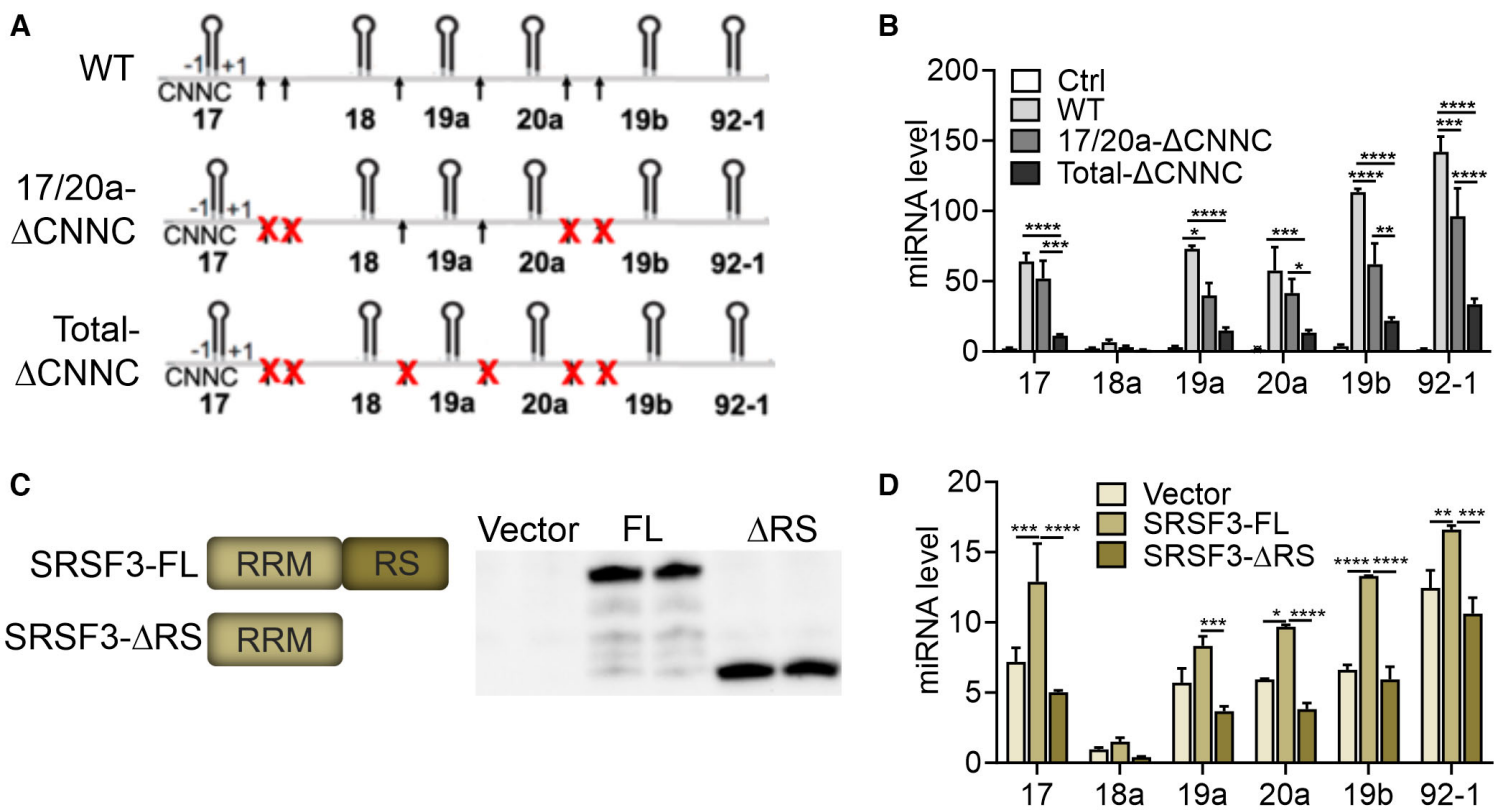
SRSF3 binding to the CNNC sites and SRSF3 RS‐domain are essential for miR‐17‐92 processing AWild‐type (WT) miR‐17‐92, 17/20a‐ΔCNNC and total‐ΔCNNC expression constructs. Synonymous mutations were introduced to remove SRSF3 binding sites (CNNC) downstream of miR‐17 and miR‐20a in 17/20a‐ΔCNNC mutant and downstream of miR‐17, miR‐18a, miR‐19a and miR‐20a in total‐ΔCNNC.BTaqMan analysis of the six miR‐17‐92 miRNAs in HEK293T cells overexpressing the WT miR‐17‐92, 17/20a‐ΔCNNC or total‐ΔCNNC construct (*P < 0.05, **P < 0.01, ***P < 0.001, ****P < 0.0001, one‐way ANOVA, mean as ± SEM, *n* = 4, biological replicates).CWestern blot analysis of HA‐tagged SRSF3‐FL or SRSF3‐ΔRS expression in LIM1215 cells (detection with a HA antibody).DTaqMan analysis of the six miR‐17‐92 miRNAs in LIM1215 cells overexpressing empty pCGN vector, SRSF3‐FL or SRSF3‐ΔRS constructs (*P < 0.05, **P < 0.01, ***P < 0.001, ****P < 0.0001, one‐way ANOVA, data as mean ± SEM, *n* = 3, biological replicates).

**Figure EV2:**
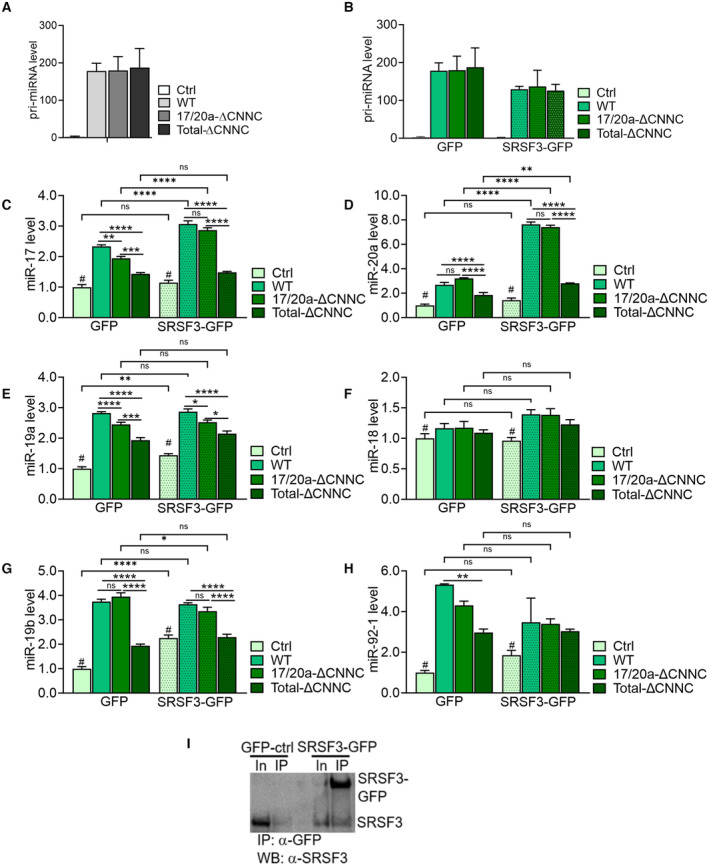
SRSF3 binding to the CNNC sites is essential for miR‐17‐92 processing ART–qPCR analysis of wild‐type (WT) miR17‐92, 17/20a‐ΔCNNC and total‐ΔCNNC construct expression in HEK293T cells (data as mean ± SEM, *n* = 4, biological replicates).BRT–qPCR analysis of wild‐type (WT) miR17‐92, 17/20a‐ΔCNNC and total‐ΔCNNC construct expression in HEK293T cells overexpressing SRSF3‐GFP or GFP control (data as mean ± SEM, *n* = 4, biological replicates).C–HTaqMan analysis of miR‐17‐92 miRNAs in HEK293 cells overexpressing SRSF3‐GFP or GFP together with WT, 17/20a‐ΔCNNC or total‐ΔCNNC miR17‐92 (*P < 0.05, **P < 0.01, ***P < 0.001, ****P < 0.0001, #all samples significantly different compared with control (Ctrl), One‐Way ANOVA, data as mean ± SEM, *n* = 4, biological replicates).ICo‐Immunoprecipitation in SRSF3‐GFP or GFP‐only expressing LIM1215 cells. In = input, IP = immunoprecipitation. The immunoprecipitation was performed with an anti‐GFP antibody and the Western blot detection with an anti‐SRSF3 antibody.

Next, we generated expression constructs carrying the wild‐type miR‐17‐92 sequence (WT), miR‐17‐92 sequence where SRSF3 CNNC‐binding sites downstream of only miR‐17 and miR‐20a (17/20a‐ΔCNNC) or all CNNC SRSF3 binding sites (total‐ΔCNNC) were mutated (Fig 2A). We overexpressed these constructs in HEK293T cells that express little endogenous miR‐17‐92 (Fig EV2A; Cloonan *et al*, 2008). The levels of miR‐17‐92‐derived miRNAs were significantly increased in cells expressing the WT construct (Fig 2B). Surprisingly, the mutation of SRSF3 CNNC‐binding sites downstream of only miR‐17 and miR‐20a (17/20a‐ΔCNNC) did not cause a selective reduction in miR‐17 and miR‐20a levels but led to a modest reduction in the levels of most miR‐17‐92‐derived miRNAs. The removal of all CNNC sites within the cluster (total‐ΔCNNC) compromised miR‐17‐92 processing by significantly decreasing the levels of all six miRNAs (Fig 2B). To verify that SRSF3 modulated miR‐17‐92 processing by binding to the CNNC sites, we moderately overexpressed SRSF3‐GFP or GFP control together with the WT, 17/20a‐ΔCNNC or total‐ΔCNNC constructs in HEK293 cells (Fig EV2B). SRSF3 overexpression selectively enhanced the processing of miR‐17 and miR‐20a in the context of WT miR‐17‐92 but not the total‐ΔCNNC mutant (Fig EV2C–D). SRSF3 overexpression had no significant effect on the processing of miR‐18a, miR‐19a/b and miR‐92‐1 in the context of either WT miR‐17‐92 or total‐ΔCNNC mutant (Fig EV2E–H). Moreover, SRSF3 depletion had no effect on miRNA processing from the total‐ΔCNNC mutant (Appendix Fig S1A–G). These data demonstrate that SRSF3 binding to the CNNC sites within miR‐17‐92 was required for the efficient processing of the cluster, SRSF3 specifically favouring the processing of two paralog miRNAs, miR‐17 and miR‐20a.

SRSF3 can interact with itself and other RS‐domain‐containing proteins via the RS‐domain (Fig EV2I; Änkö *et al*, 2010). To investigate whether interactions via RS‐domains could play a role in the processing of miRNAs from the miR‐17‐92 cluster, we robustly overexpressed full‐length SRSF3 and SRSF3 without the RS‐domain (CMV‐driven SRSF3‐FL and SRSF3‐ΔRS, respectively) in LIM1215 cells that express endogenous miR‐17‐92 (Fig 2C). The high overexpression of SRSF3‐FL led to an increase in mature miRNA levels across the cluster (Fig 2D). A similar level of overexpression of SRSF3‐ΔRS failed to enhance the mature miRNA levels demonstrating that SRSF3 RS‐domain was essential for SRSF3 functions in miR‐17‐92 processing (Fig 2D). Taken together, these data demonstrate that SRSF3 binding to multiple sites within the miR‐17‐92 transcript and the SRSF3 RS‐domain are essential for the efficient processing of the miR‐17‐92 cluster.

### SRSF3 binding rearranges the secondary structure of miR‐17‐92

**Figure 3:**
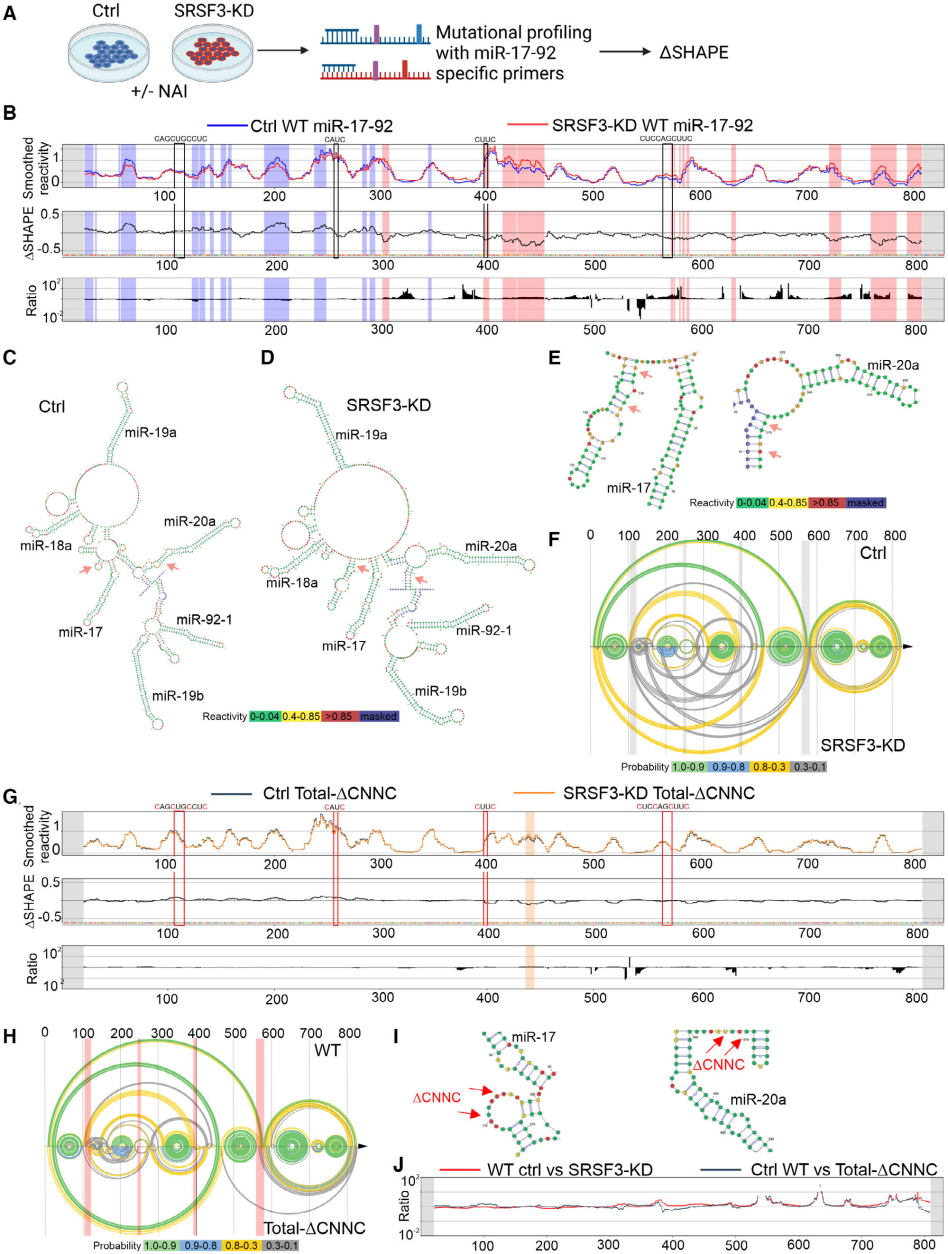
SRSF3 binding rearranges the secondary structure of miR‐17‐92 ASchematic of in‐cell SHAPE‐MaP in SRSF3‐KD and control cells.BVisualisation and comparison of WT miR‐17‐92 SHAPE reactivities in control and SRSF3‐KD cells. *First panel*: Mean control (blue) and SRSF3‐KD (red) SHAPE reactivities, calculated over 11 nt sliding windows. *Second panel*: ΔSHAPE reactivity differences between control and SRSF3‐KD. Regions with higher reactivity in control are marked with blue shading and regions with higher reactivity in SRSF3‐KD with red shading. *Third panel*: The SRSF3‐KD to control reactivity ratios. The black boxes mark the CNNC sites.C, DSecondary structure prediction of WT miR‐17‐92 in control (C) and SRSF3‐KD (D) cells based on SHAPE reactivities. The secondary structure was modelled with SuperFold and visualised using VARNA. The pink arrows mark CNNC regions downstream of miR‐17/20a.EThe predicted RNA secondary structure around SRSF3 binding CNNC sites downstream of miR‐17 and miR‐20a stem‐loops in SRSF3‐KD cells. The pink arrows mark the CNNC regions.FArc plot depicting base pairing probabilities within WT miR‐17‐92 in control and SRSF3‐KD cells.GVisualisation and comparison of total‐ΔCNNC miR‐17‐92 SHAPE reactivities in control and SRSF3‐KD cells. *First panel*: Mean control (grey) and SRSF3‐KD (orange) SHAPE reactivities, calculated over 11 nt sliding windows. *Second panel*: ΔSHAPE reactivity differences between control and SRSF3‐KD. Regions with higher reactivity in control are marked with grey shading and regions with higher reactivity in SRSF3‐KD with orange shading. *Third panel*: The SRSF3‐KD to control reactivity ratios. The red boxes mark the mutated CNNC sites.HArc plot depicting base pairing probabilities within WT and total‐ΔCNNC miR‐17‐92 in control cells.IThe predicted total‐ΔCNNC miR‐17‐92 secondary structure around the mutated CNNC sites downstream of miR‐17 and miR‐20a. The red arrows mark the mutated CNNC regions.JThe WT miR‐17‐92 in SRSF3‐KD (red) to total‐ΔCNNC miR‐17‐92 in control (grey) reactivity ratios.

**Figure EV3:**
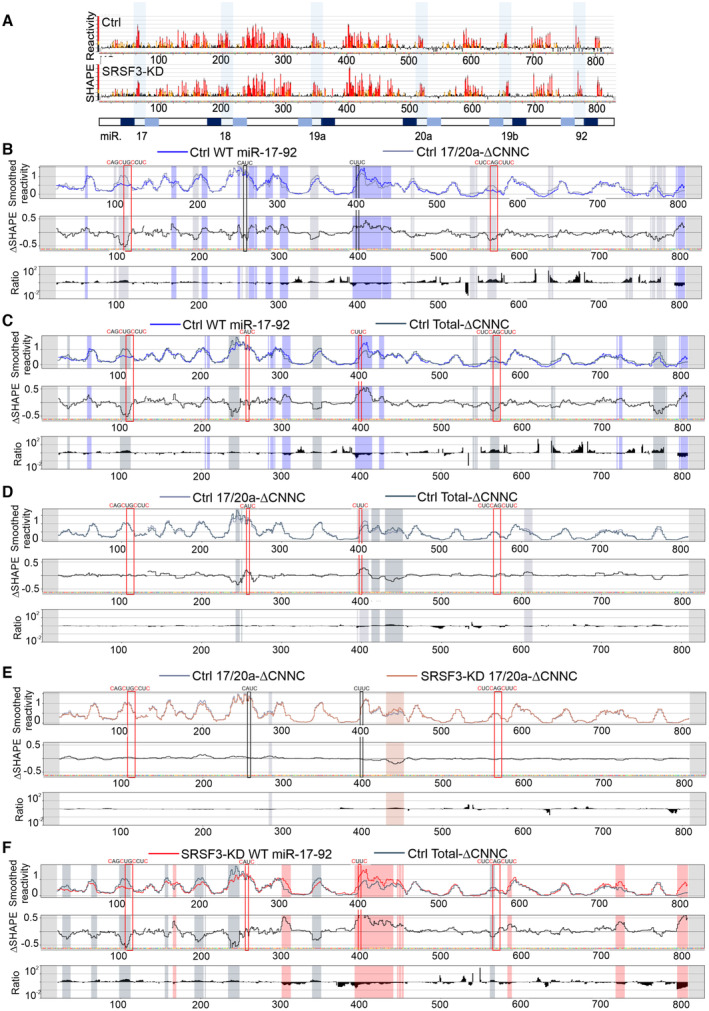
SRSF3 rearranges the miR‐17‐92 secondary structure through CNNC sites AWT miR‐17‐92 SHAPE reactivities in control (Ctrl) and SRSF3‐KD cells based on ShapeMapper2. Red: SHAPE≥0.85, Yellow: 0.85 > SHAPE > 0.4 and Black: SHAPE ≤ 0.4. The bar below represents miR‐17‐92 transcript with the miRbase predicted miRNA active strands marked in dark blue and star strands in light blue.B, CComparison of 17/20a‐ΔCNNC (B) and total‐ΔCNNC (C) miR‐17‐92 SHAPE reactivities to WT miR‐17‐92 SHAPE reactivities. *First panel*: Mean reactivities, calculated over 11 nt sliding windows. *Second panel*: ΔSHAPE reactivity differences between WT and 17/20a‐ΔCNNC or total‐ΔCNNC miR‐17‐92. Regions with higher reactivity in WT miR‐17‐92 are marked with blue shading and regions with higher reactivity in 17/20a‐ΔCNNC or total‐ΔCNNC with grey shading. *Third panel*: The 17/20a‐ΔCNNC to WT miR‐17‐92 or total‐ΔCNNC to WT miR‐17‐92 reactivity ratios. The boxes mark CNNC sites (black WT, red CNNC sites mutated).DComparison of miR‐17‐92 SHAPE reactivities between 17/20a‐ΔCNNC and total‐ΔCNNC miR‐17‐92. *First panel*: Mean reactivities, calculated over 11 nt sliding windows. *Second panel*: ΔSHAPE reactivity differences between 17/20a‐ΔCNNC and total‐ΔCNNC miR‐17‐92. Regions with higher reactivity in 17/20a‐ΔCNNC are marked with light grey shading and regions with higher reactivity in total‐ΔCNNC miR‐17‐92 are marked with dark grey shading. *Third panel*: The total‐ΔCNNC to 17/20a‐ΔCNNC reactivity ratios. The red boxes mark the mutated CNNC sites.EVisualisation and comparison of 17/20a‐ΔCNNC miR‐17‐92 SHAPE reactivities in control and SRSF3‐KD cells. *First panel*: Mean control (grey) and SRSF3‐KD (brown) SHAPE reactivities, calculated over 11 nt sliding windows. *Second panel*: ΔSHAPE reactivity differences between control and SRSF3‐KD. Regions with higher reactivity in control are marked with grey shading and regions with higher reactivity in SRSF3‐KD with brown shading. *Third panel*: The SRSF3‐KD to control reactivity ratios. The boxes mark CNNC sites (black WT, red CNNC sites mutated).FVisualisation and comparison of WT miR‐17‐92 SHAPE reactivities in SRSF3‐KD cells and total‐ΔCNNC miR‐17‐92 SHAPE reactivities in control cells. *First panel*: Mean WT miR‐17‐92 SRSF3‐KD (red) and total‐ΔCNNC miR‐17‐92 control (grey) SHAPE reactivities, calculated over 11 nt sliding windows. *Second panel*: ΔSHAPE reactivity differences between WT miR‐17‐92 SRSF3‐KD and total‐ΔCNNC miR‐17‐92 control. Regions with higher reactivity in WT miR‐17‐92 SRSF3‐KD are marked with red shading and regions with higher reactivity in total‐ΔCNNC miR‐17‐92 control with grey shading. *Third panel*: The total‐ΔCNNC miR‐17‐92 control to WT miR‐17‐92 SRSF3‐KD reactivity ratios. The red boxes mark the mutated CNNC sites.

Modelling and *in vitro* studies have shown that miR‐17‐92 assumes a secondary structure that needs to be disrupted to make miRNA cleavage sites accessible (Chakraborty & Krishnan, 2017). As both SRSF3 binding to multiple sites within miR‐17‐92 and the RS‐domain were required for the efficient processing, SRSF3 could mediate its effects by altering the structure of the polycistron. To determine whether SRSF3 binding affected the miR‐17‐92 secondary structure in cells, we performed target specific in‐cell SHAPE‐MaP analysis in SRSF3 knockdown (SRSF3‐KD) and control cells (Fig 3A), probing the reactivity of each nucleotide within miR‐17‐92 RNA to NAI (2‐methylnicotinic acid imidazolide; Smola & Weeks, 2018). The sites of chemical modification‐induced mutations were identified using the ShapeMapper software (Appendix Table S3; Busan & Weeks, 2018). We detected strong reactivities indicative of flexible nucleotides in the loop regions and low reactivities in the stem regions of the individual miRNA stem‐loops in the control and SRSF3‐KD cells (Figs 3B and EV3A). The *in vivo* miR‐17‐92 secondary structure predicted based on the SHAPE reactivities markedly differed from the *in silico* predicted minimum free‐energy structure (Fig 3C; Appendix Fig S2A), showing that cellular interactions superseded simple thermodynamic properties of the nucleotides. The comparison of control (Fig 3B, blue line) and SRSF3‐KD (Fig 3B, red line) reactivities by ΔSHAPE revealed significant differences across the RNA, identifying both regions with significantly higher reactivities in the control (Fig 3B, blue shaded regions) and regions where SRSF3‐KD reactivities were higher compared with control (Fig 3B, red shaded regions). The SRSF3‐KD to control reactivity ratios (Fig 3B, ratio) suggested the reactivity differences reflected alterations in RNA protein binding (Smola & Weeks, 2018). The reactivity differences were not limited to the CNNC sites, but SRSF3 depletion resulted in broad changes in the miR‐17‐92 SHAPE reactivities (Fig 3B). The miR‐17‐92 secondary structures predicted based on the SHAPE reactivities in the presence and absence of SRSF3 further demonstrated that SRSF3 binding to the CNNC sites modulated both local and distant base pairing (Fig 3C–F). In the absence of SRSF3, the CNNC sites downstream of miR‐17 and miR‐20a were in stem structures while in the control cells they were in less structured regions (Fig 3E and F), suggesting that SRSF3 binding disrupts base pairing around the CNNC sites. The alterations in distant base pairing probabilities across miR‐17‐92 RNA following SRSF3 depletion further suggested that SRSF3 binding to the CNNC sites significantly modulated the global structure of the miR‐17‐92 polycistron (Fig 3F).

To further demonstrate that SRSF3 modulated miR‐17‐92 structure by binding to the CNNC sites, we conducted in‐cell SHAPE‐MaP analysis of 17/20a‐ΔCNNC and total‐ΔCNNC mutants in SRSF3‐depleted and control cells. Based on *in silico* minimum free‐energy structure predictions, the introduced point mutations altered the thermodynamical properties of miR‐17‐92 RNA (Appendix Fig S2A–C). Accordingly, the comparison of in‐cell SHAPE reactivities of WT, 17/20a‐ΔCNNC and total‐ΔCNNC miR‐17‐92 in control cells demonstrated significant reactivity differences at the mutation sites (Fig EV3B–D). Importantly, SRSF3 depletion had no effect on the total‐ΔCNNC miR‐17‐92 reactivity profile and affected the 17/20a‐ΔCNNC miR‐17‐92 reactivities only downstream of the two CNNC sites that were not mutated, demonstrating that SRSF3 modulated miR‐17‐92 structure by binding to the CNNC sites (Figs 3G and EV3E). Similar to SRSF3 depletion, the introduction of CNNC mutations rearranged the miR‐17‐92 secondary structure beyond local base pairing interactions at the CNNC sites (Fig 3H; Appendix Fig S3A–C). While the CNNC sites downstream of miR‐17/20a base paired following SRSF3 depletion (Fig 3E), in total‐ΔCNNC miR‐17‐92 these regions were single‐stranded because of lost nucleotide complementarity due to the CNNC mutations (Fig 3I). However, the effects of SRSF3 depletion and SRSF3 binding site mutations on the SHAPE reactivity ratios reflecting alterations in RNA protein binding were remarkably similar (Figs 3J and EV3F), suggesting that in both cases the local changes around the CNNC sites resulted in global structure rearrangements.

Taken together, the SHAPE‐MaP data demonstrated that miR‐17‐92 secondary structure could be modulated either by SRSF3 depletion or SRSF3 binding site mutations. SRSF3 binding within miR‐17‐92 RNA disrupted base pairing of the CNNC regions and, thereby, rearranged the miR‐17‐92 secondary structure. The SRSF3‐mediated structural changes may alter the access of the Microprocessor complex to miR‐17‐92 cleavage sites, potentially favouring some sites over others. The dependence of miR‐17‐92 processing on the SRSF3 RS‐domain further suggests that RS‐domain interactions across the CNNC sites may contribute to the accessibility of Drosha cleavage sites.

### SRSF3 controls mouse and human CDKN1A levels through miR‐17/20a

**Figure 4:**
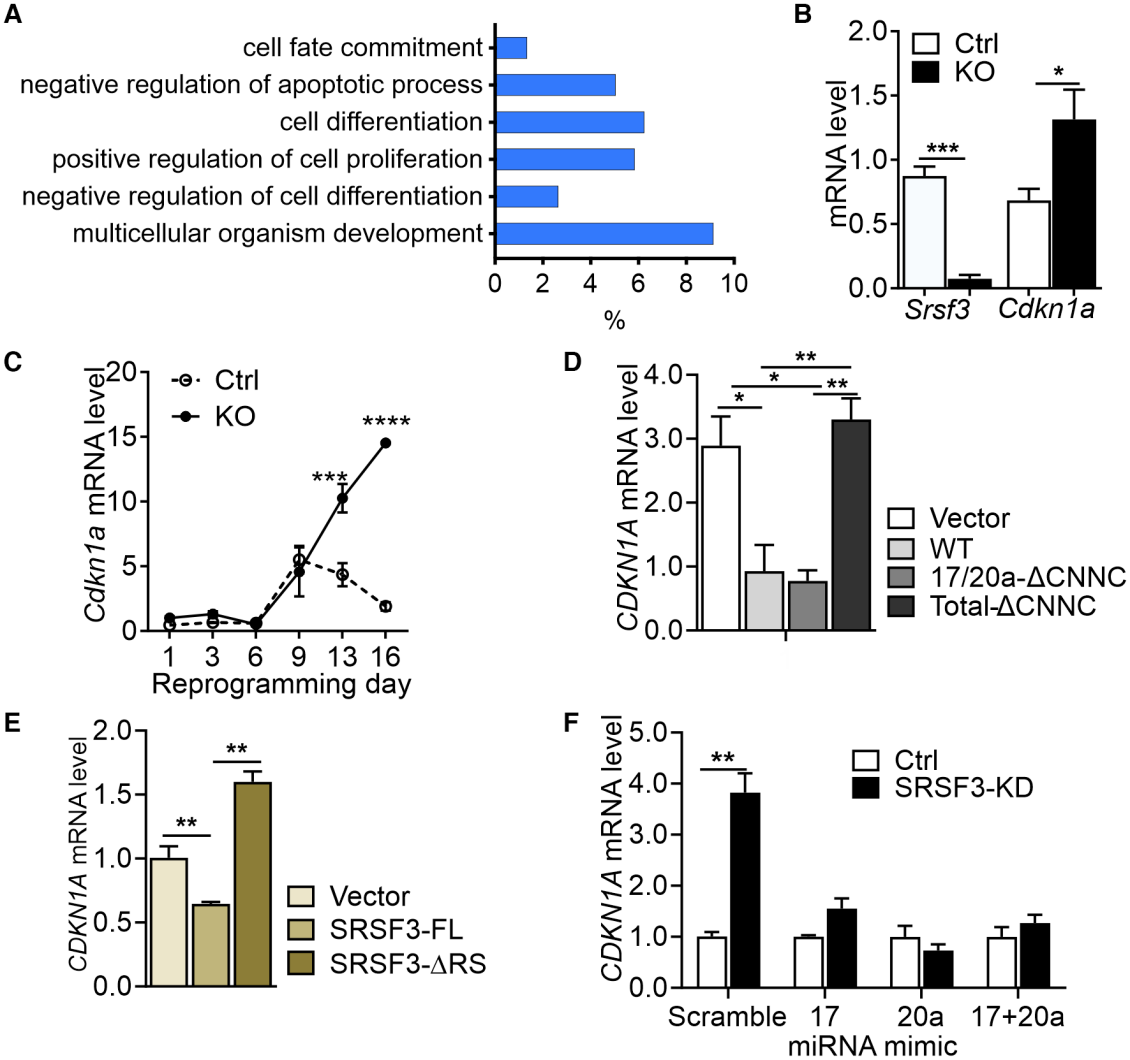
SRSF3 controls mouse and human CDKN1a levels through miR‐17/20a AEnriched GO terms of differentially expressed genes following SRSF3 depletion in mouse iPSCs. The RNA‐sequencing data are based on (Ratnadiwakara *et al*, 2018).BRT–qPCR quantification of *Srsf3* and *Cdkn1a* (p21) mRNA levels in *Srsf3*‐knockout (KO) and control (Ctrl) iPSCs (*P < 0.05, *** < 0.001, two‐tailed unpaired Student's *t*‐test, data as mean ± SEM, *n* = 3, biological replicates).CQuantification of *Cdkn1a* mRNA expression by RT–qPCR during reprogramming in *Srsf3*‐KO and control cells (***P < 0.001, ****P < 0.0001, Two‐way ANOVA, data as mean ± SEM, *n* = 3, biological replicates).DRT–qPCR quantification of *CDKN1A* (p21) levels in HEK293T cells overexpressing empty vector, WT miR‐17‐92, 17/20a‐ΔCNNC and total‐CNNCΔ expression constructs (*P < 0.05, **P < 0.01, One‐way ANOVA, data as mean ± SEM, *n* = 3, biological replicates).ERT–qPCR quantification of *CDKN1A* (p21) levels in LIM1215 cells overexpressing empty vector, SRSF3‐FL and SRSF3‐ΔRS constructs (**P < 0.01, One‐way ANOVA, data as mean ± SEM, *n* = 3, biological replicates).FRT–qPCR quantification of *CDKN1A* (p21) levels in LIM1215 cells overexpressing scrambled control, miR‐17, miR‐20a or miR‐17 + miR‐20a mimics (**P < 0.01, One‐way ANOVA, data as mean ± SEM, *n* = 3, biological replicates).

**Figure EV4:**
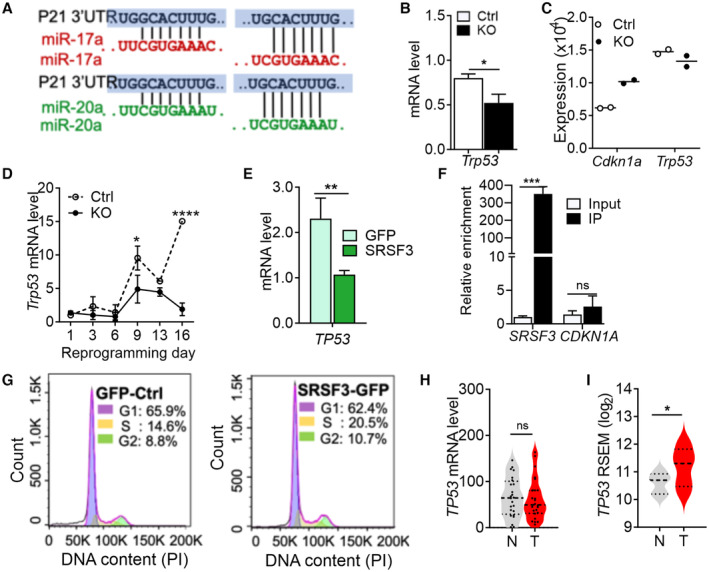
SRSF3 controls CDKN1a/p21 levels through miR‐17/20a independent of p53 ASchematic of miR‐17 and miR‐20a seed regions that target the *Cdkn1a* 3′UTR at two sites.BRT–qPCR quantification of *Trp53* (p53) mRNA levels in *Srsf3*‐knockout (KO) and control (Ctrl) iPSCs (*P < 0.05, two‐tailed unpaired Student's *t*‐test, data as mean ± SEM, *n* = 3, biological replicates).CQuantification of *Cdkn1a* and *Trp53* expression from *Srsf3*‐KO and Ctrl RNA‐sequencing data from (Ratnadiwakara *et al*, 2018; data as mean, *n* = 2, biological replicates).DQuantification of *Trp53* mRNA expression by RT–qPCR during reprogramming in *Srsf3*‐KO (KO) and control (Ctrl) cells (*P < 0.05, ****P < 0.0001, two‐way ANOVA, data as mean ± SEM, *n* = 3, biological replicates).ERT–qPCR quantification of *TP53* (p53) mRNA levels in SRSF3 overexpressing (SRSF3‐GFP) and control (GFP) LIM1215 cells (**P < 0.01, two‐tailed unpaired Student's *t*‐test, data as mean ± SEM, *n* = 3, biological replicates).FRNA immunoprecipitation (IP) of *SRSF3* (positive control) and *CDKN1A* mRNAs in GFP‐only and SRSF3‐GFP expressing LIM1215 cells (***P < 0.001, Two‐tailed unpaired Student's *t*‐test, data as mean ± SEM, *n* = 3, biological replicates).GRepresentative histograms of propidium iodide‐stained control (GFP) and SRSF3‐T2A‐GFP overexpressing (SRSF3‐GFP) LIM1215 cells analysed by flow cytometry. Histograms were generated using cell cycle analysis tool in FlowJo software (Tree Star). Per cent cells in each phase are indicated in the inset.HRT–qPCR quantification of *TP53* mRNA levels in colorectal tumours and their paired normal samples (ns = not significant, two‐tailed unpaired Student's *t*‐test, *n* = 25, biological replicates, central band = median, dotted lines = 25^th^ and 75^th^ percentile).IRelative *TP53* expression in TCGA COAD data (version 2016_01_28, tumour *n* = 459 and normal *n* = 41, *P < 0.05, two‐tailed unpaired Student's *t*‐test, biological replicates, central band = median, dotted lines = 25^th^ and 75^th^ percentile).

SRSF3 plays a dual role in mouse pluripotent cells by enhancing the establishment and maintenance of the pluripotent state and promoting cell cycle and proliferation (Ratnadiwakara *et al*, 2018). Accordingly, SRSF3 depletion did not only affect the pluripotency network genes but also the expression of genes involved in the control of cell proliferation (Fig 4A; Ratnadiwakara *et al*, 2018). Since miR‐17‐92‐derived miRNAs promote proliferation and inhibit apoptosis (Spaccarotella *et al*, 2014; Zhou *et al*, 2014), we investigated whether SRSF3 controlled cell proliferation in pluripotent cells through the miR‐17‐92 cluster miRNAs. The paralogs, miR‐17 and miR‐20a, that were downregulated following SRSF3 depletion are known to promote cell cycle progression and cell proliferation in pluripotent cells (Cloonan *et al*, 2008; Wang *et al*, 2008). The cell cycle regulator, cyclin‐dependant kinase inhibitor *Cdkn1a* (p21) is a *bona fide* target of miR‐17/miR‐20a (Mendell, 2008; Olive *et al*, 2013; Agarwal *et al*, 2015). The 3′UTR of *Cdkn1a* shares a perfect complementarity with the miR‐17/20a seed regions (Fig EV4A) leading to mRNA destabilisation and/or translational repression (Iwakawa & Tomari, 2022). The degradation of *CDKN1A*/*Cdkn1a* mRNA due to miR‐17 or miR‐20a activity has been previously reported in different cellular contexts (Wong *et al*, 2010; Chen *et al*, 2016; Zhou *et al*, 2021). Accordingly, RNA‐sequencing and RT–qPCR analysis demonstrated increased *Cdkn1a* mRNA expression in SRSF3‐depleted mouse pluripotent cells independent of *Trp53* levels (Figs 4B and EV4B and C). In fact, *Trp53* levels were downregulated, in accordance with a previous study reporting a p53‐independent regulation of p21 in ES cells (Suvorova *et al*, 2016). During reprogramming from mouse embryonic fibroblasts (MEFs) into induced pluripotent stem (iPS) cells, *Cdkn1a* levels followed *Trp53* levels until reprogramming day 13 when their expression diverged as the cells committed to the pluripotent fate (Figs 4C and EV4D). *Cdkn1a* expression was affected by SRSF3 depletion only after Day 13 of reprogramming when miR‐17‐92 expression was turned on (Polo *et al*, 2012). The miR‐17/20a target *CDKN1A* expression was significantly downregulated also in HEK293 cells expressing the miR‐17‐92 WT construct while the expression level was not affected in cells expressing total‐ΔCNNC construct (Fig 4D). In cells expressing 17/20a‐ΔCNNC construct, *CDKN1A* levels were similar to cells expressing the WT construct, in line with only a small reduction in miR‐17/20a levels (Fig 4D). In cells robustly overexpressing SRSF3‐FL, *CDKN1A* levels were downregulated while the overexpression of SRSF3‐ΔRS abolished this effect (Fig 4E). The introduction of a miR‐17 or miR‐20a mimics partially rescued *CDKN1A* levels, while the combination of both mimics completely restored *CDKN1A* expression in SRSF3‐depleted cells (Fig 4F). In conclusion, SRSF3‐mediated processing of miR‐17 and miR‐20a resulted in downregulation of a key miR‐17/20a downstream target *Cdkn1a*/*CDKN1A* in both mouse pluripotent and human HEK293 cells.

### SRSF3 regulates cell proliferation and cell cycle through miR‐17/20a‐CDKN1A in human colorectal cancer cells

**Figure 5:**
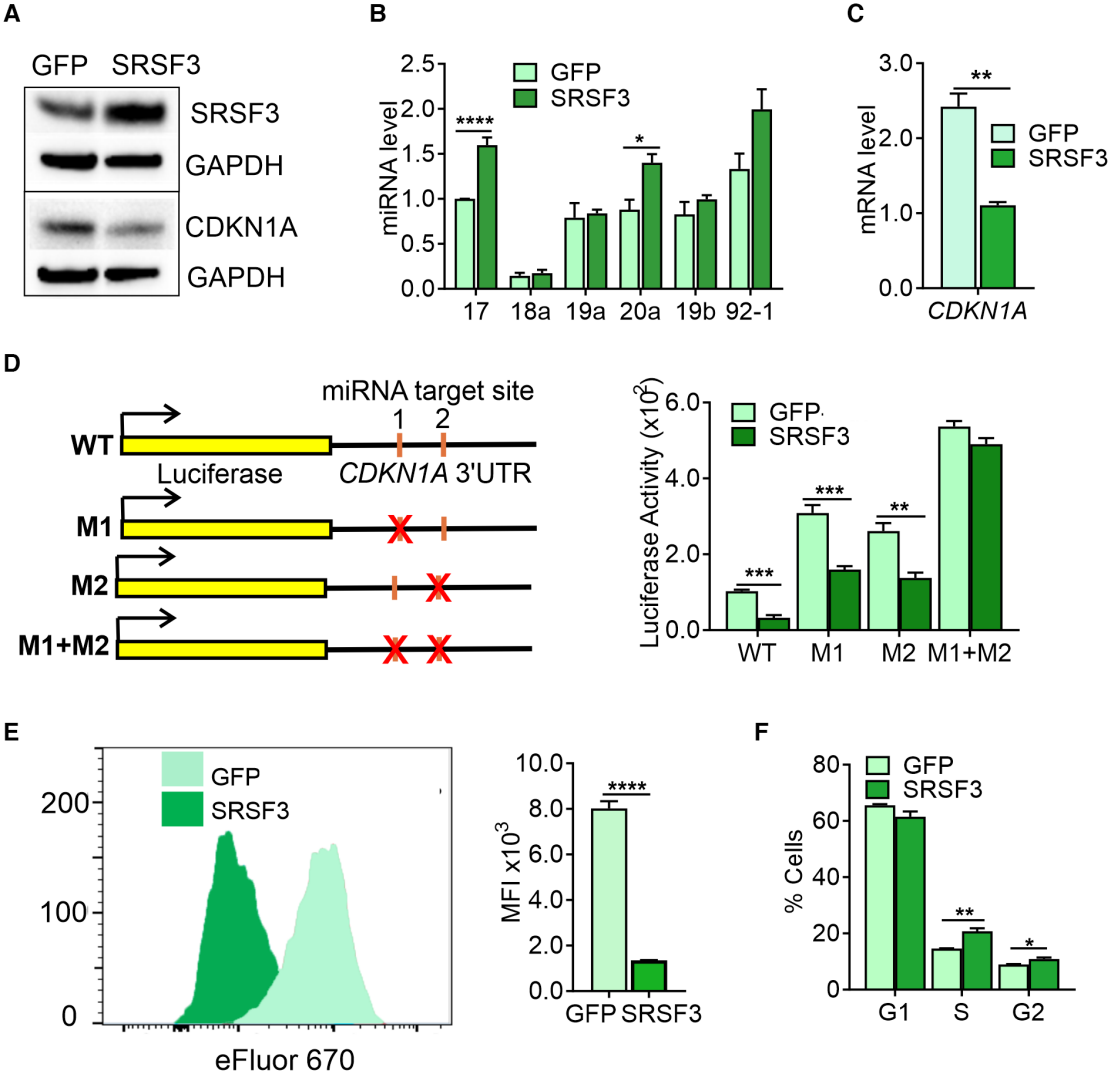
SRSF3 regulates cell proliferation and cell cycle through miR‐17/20a‐CDKN1A in human colorectal cancer cells AWestern blot analysis of SRSF3 and CDKN1A expression in SRSF3‐T2A‐GFP overexpressing (SRSF3) and control (GFP) LIM1215 cells. GAPDH served as a loading control.BTaqMan analysis of miR‐17‐92 miRNAs in SRSF3‐T2A‐GFP overexpressing (SRSF3‐GFP) and GFP‐only control (GFP) LIM1215 cells (*P < 0.05, ****P < 0.001, two‐tailed unpaired Student's *t*‐test, data as mean ± SEM, *n* = 4, biological replicates).CRT–qPCR quantification of *CDKN1A* (p21) mRNA levels in SRSF3‐T2A‐GFP overexpressing (SRSF3‐GFP) and control (GFP) LIM1215 cells (**P < 0.01, two‐tailed unpaired Student's *t*‐test, data as mean ± SEM, *n* = 3, biological replicates).D
*Left*: Schematic of the Firefly luciferase reporter constructs (Wang *et al*, 2008). *Right*: Luciferase activity in SRSF3 overexpressing (SRSF3‐GFP) and control (GFP) LIM1215 cells transfected with the reporter constructs (**P < 0.01, ***P < 0.001, two‐tailed unpaired Student's *t*‐test, data as mean ± SEM, *n* = 4, biological replicates).EA representative histogram and quantification of mean fluorescent intensity (MFI) of eFluor 670 labelled SRSF3 overexpressing (SRSF3‐GFP) and control (GFP) LIM1215 cells (****P < 0.0001, two‐tailed unpaired Student's *t*‐test, data as mean ± SEM, *n* = 3, biological replicates).FQuantification of fraction of cells in G1, S and G2 phases in SRSF3 overexpressing cells (SRSF3‐GFP) relative to control (GFP) LIM1215 cells (*P < 0.05, **P < 0.01, two‐tailed unpaired Student's *t*‐test, data as mean ± SEM, *n* = 4, biological replicates).

The miRNAs produced from the cluster are frequently dysregulated in various cancers (Ma *et al*, 2012; Fu *et al*, 2018; Xu *et al*, 2019a), and miR‐17‐92 was one of the first identified miRNA oncogenes (aka OncomiR‐1; Hayashita *et al*, 2005; He *et al*, 2005; Mendell, 2008; Yu *et al*, 2012). SRSF3 is a proto‐oncogene able to enhance cell cycle progression and cell proliferation in both normal and cancer cells, including CRC (Kuranaga *et al*, 2018; Wang  *et al*, 2020). We overexpressed SRSF3 together with a GFP reporter in the human CRC line LIM1215 that endogenously expresses miR‐17‐92 (Fig 5A). The level of SRSF3 overexpression in LIM1215 cells corresponded to the levels observed in primary tumours. SRSF3 overexpression led to increased levels of miR‐17 and miR‐20a (Fig 5B) and to a decrease in *CDKN1A*/p21 mRNA and protein levels without a change in *TP53* levels that was expressed at a low level in LIM1215 cells (Figs 5A and C, and EV4E). RNA immunoprecipitation in LIM1215 cells overexpressing SRSF3‐GFP showed that SRSF3 does not directly interact with *CDKN1A* mRNA (Fig EV4F), and a *CDKN1A* 3′UTR luciferase reporter assay (Wang *et al*, 2008) further demonstrated that SRSF3 regulated *CDKN1A* via miR‐17/20a in human CRC cells (Fig 5D).

In accordance with the reduced *CDKN1A* levels and similar to mouse pluripotent cells and other human cell lines (Jia *et al*, 2010; Ratnadiwakara *et al*, 2018), SRSF3 overexpression enhanced cell proliferation and G1‐to‐S transition of LIM1215 cells (Figs 5E and F, and EV4G; Savatier *et al*, 1994; Becker *et al*, 2006; Fluckiger *et al*, 2006; Bartel, 2018; Ratnadiwakara *et al*, 2018). CDKN1A belongs to the CyclinE/CDK2 pathway consisting of multiple cell cycle inhibitors, many of which are predicted miR‐17/20a targets (Backes *et al*, 2017). Among the 16 cell cycle‐associated genes analysed, only *CDKN1A* expression was increased more than 1.5‐fold in SRSF3‐depleted cells (Appendix Table S4), suggesting that the cell proliferation and cell cycle phenotype in human cancer cells was largely mediated through miR‐17a/20a targeting *CDKN1A*.

### SRSF3‐miR‐17/20a‐CDKN1A pathway marks advanced colorectal cancer

**Figure 6:**
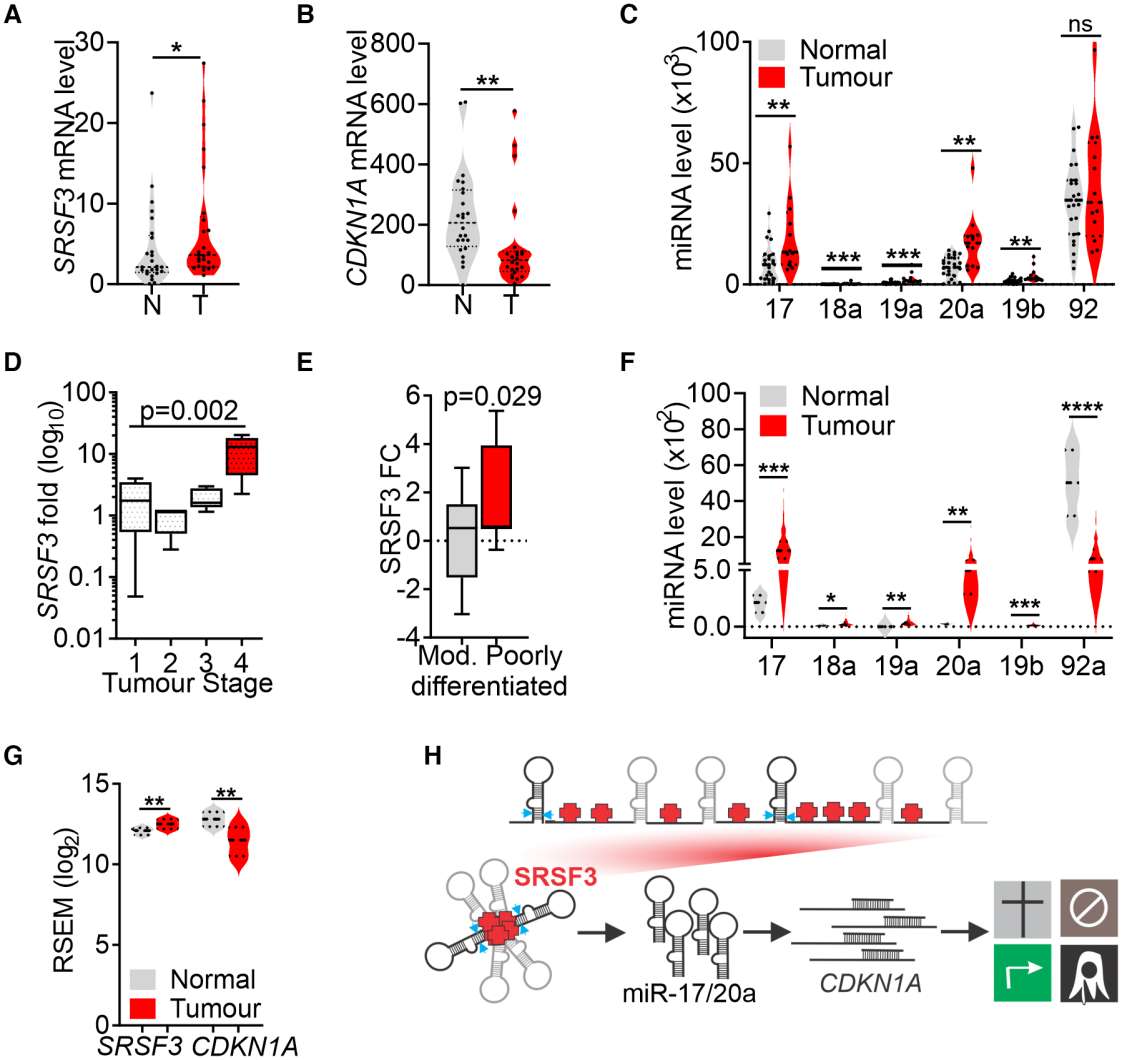
SRSF3‐miR‐17/20a‐CDKN1A pathway marks advanced colorectal cancer A, BRT–qPCR quantification of *SRSF3* (A) and *CDKN1A* (B) mRNA levels in colorectal tumours and their paired normal samples (*P < 0.05, **P < 0.01, two‐tailed unpaired Student's *t*‐test, data as mean ± SEM, *n* = 25, biological replicates). N = normal, T = tumour.CTaqMan analysis of miR‐17‐92 miRNAs in colorectal tumours and their paired normal samples (**P < 0.01, ***P < 0.001, two‐tailed unpaired Student's *t*‐test, data as mean ± SEM, *n* = 25, biological replicates).DAssociation between *SRSF3* expression and tumour (T) stage. T1 (*n* = 3), T2 (*n* = 5), T3 (*n* = 11) and T4 (*n* = 3) tumours, *SRSF3* fold change as the dependent variable. Linear regression analysis (central band = median, boxes = 25^th^ and 75^th^ percentile and whiskers = min and max, *n* = biological replicates).EAssociation between *SRSF3* expression and tumour differentiation status. Moderately (Mod.) differentiated tumours (*n* = 19) and poorly differentiated tumours (*n* = 5), *SRSF3* fold change as the dependent variable. Linear regression analysis (central band = median, boxes = 25^th^ and 75^th^ percentile and whiskers = min and max; *n* = biological replicates).FExpression of miR‐17‐92 cluster miRNAs in TCGA COAD data (version 2016_01_28; tumour *n* = 272 and normal *n* = 8 (biological replicates); RSEM, RNA‐seq by Expectation Maximation; *P < 0.05, **P < 0.01, ***P < 0.001, ****P < 0.0001, two‐tailed Unpaired Student's *t*‐test, central band = median, dotted lines = 25^th^ and 75^th^ percentile).G
*SRSF3* and *CDKN1A* expression in TCGA COAD data (version 2016_01_28; tumour *n* = 459 and normal *n* = 41 (biological replicates); RSEM, RNA‐seq by Expectation Maximation; **P < 0.01, two‐tailed unpaired Student's *t*‐test, central band = median, dotted lines = 25^th^ and 75^th^ percentile).HA model for SRSF3‐mediated control of miR‐17‐92 structure that enables the enhanced processing of miR‐17 and miR‐20 and ultimately promotes the self‐renewal properties of colorectal cancer cells, suggesting a direct link between SRSF3‐mediated pri‐miRNA structure and cancer pathogenesis.

Finally, we investigated whether SRSF3‐regulated miR‐17‐92 processing played a role in human CRC patients. Analysis of tumour and adjacent normal tissue from 25 CRC patients not subjected to chemo‐ or radiotherapy demonstrated significantly increased *SRSF3* mRNA levels in the tumour compared with their paired normal tissue (Fig 6A). Similar to mouse induced pluripotent cells (Fig 4B), *CDKN1A* mRNA levels anticorrelated with *SRSF3* mRNA levels in tumour samples independent of *TP53* levels (Figs 6B and EV4H). Importantly, miRNAs of the miR‐17‐92 cluster were significantly upregulated in tumour samples with particularly high levels of miR‐17 and miR‐20a (Fig 6C; Appendix Table S5). Sixty per cent of the tumours analysed displayed this signature of *SRSF3*‐miR‐17/20a‐*CDKN1A* expression. *SRSF3* mRNA expression was significantly associated with the T4 stage (advanced cancer with tumour penetrating through all layers of colon and possibly into adjacent organs) compared with T1 stage tumours (*P* = 0.002; Fig 6D) and with poorly differentiated tumours (high grade/anaplastic) compared with moderately differentiated (intermediate grade) tumours (*P* = 0.029; Fig 6E), in agreement with the role of SRSF3 in self‐renewal (Ratnadiwakara *et al*, 2018).

We also analysed TCGA COAD data, including mRNA expression data for 459 tumour and 41 normal samples and miRNA expression data for 272 tumour and eight normal samples (version 2016_01_28) for *SRSF3*‐miR‐17/20a‐*CDKN1A* expression signature. The TCGA data supported our initial patient cohort data with an increase in *SRSF3* expression, downregulation of *CDKN1A* levels and upregulation of miRNAs of the miR‐17‐92 cluster in tumour samples compared with normal tissue, with the largest increase in miR‐17 and miR‐20a levels (Fig 6F and G). While no difference in *TP53* levels was observed in our patient cohort, in the larger TCGA cohort *TP53* levels were increased (Fig EV4I) similar to pluripotent cells (Fig EV4B). In conclusion, these data suggest that SRSF3 acts as an upstream regulator of *CDKN1A* mRNA expression through miR‐17 and miR‐20a both in normal self‐renewing cells and in the context of CRC. Thus, SRSF3‐mediated alterations in miR‐17‐92 processing leading to increased levels of oncogenic miRNAs that promote self‐renewal properties of tumour cells may play a key role in CRC progression.

## Discussion

Here, we uncover that SRSF3 enhances the processing of the miR‐17‐92 polycistron and rearranges its secondary structure. We demonstrate that both SRSF3 binding to multiple CNNC sites within miR‐17‐92 and SRSF3 RS‐domain are critical for efficient miR‐17‐92 processing. SRSF3 binding to CNNC sites disrupts base pairing interactions within the miR‐17‐92, resulting in global secondary structure changes. In mouse pluripotent and human colorectal cancer cells, SRSF3 binding enhances the processing of two paralog miRNAs, miR‐17 and miR‐20, that control cell cycle and proliferation through CDKN1A/p21. The conserved SRSF3‐miR‐17/20a‐CDKN1A pathway operates in human CRC patients, linking the miR‐17‐92 processing facilitated by SRSF3 to pathogenic mechanisms of disease. Our work demonstrates the potential of RNA binding proteins in shaping *in vivo* RNA structures to control key cellular functions.

The SRSF3 consensus binding site CNNC and/or experimentally defined SRSF3 binding sites are found downstream of Drosha cleavage sites in the majority of mouse and human pri‐miRNAs (Auyeung *et al*, 2013). The binding of SRSF3 at 17‐18 nt downstream of the Drosha cleavage site has been shown to enhance the Drosha cleavage of multiple pri‐miRNA hairpins, including miR‐16, miR‐17, miR‐20a, miR‐30a/c and miR‐142, *in vitro* (Auyeung *et al*, 2013; Fernandez *et al*, 2017; Kim *et al*, 2018). SRSF3 has been proposed to recruit Drosha to the basal junction of pri‐miRNAs; however, a direct protein–protein interaction between SRSF3 and Drosha was not observed (Kim *et al*, 2018). SRSF3 binding was shown to suppress unproductive 5′ nick and reverse processing of numerous pri‐miRNAs *in vitro*, most notably the miR‐17 family miRNAs, potentially by stabilising the Microprocessor‐RNA complex or guiding the Microprocessor to the right orientation at the basal junction (Kim *et al*, 2021). Our data demonstrate that following SRSF3 depletion, SRSF3 target sites downstream of miR‐17 and miR‐20a base paired in stem structures. In addition, new distant base pairing interactions were formed in the absence of SRSF3. Similarly, CNNC site mutations led to global changes in miR‐17‐92 secondary structure. SRSF3 depletion had no effect on the structure of the miR‐17‐92 mutant lacking CNNC sites. Thus, our data demonstrating a change in base pairing and miR‐17‐92 structure upon SRSF3 binding to the CNNC sites propose a model where SRSF3 may favour the access of the Microprocessor complex to distinct cleavage sites by constraining the RNA structure (Fig 6H). The G‐to‐A mutation in the cancer‐associated variant of pri‐miR‐30c‐1 leads to a change in the pri‐miR‐30c‐1 structure enabling SRSF3 binding (Fernandez *et al*, 2017). Recent study demonstrated that SRSF3 can bind to CNNC sites in both single‐ and double‐stranded regions within the miR‐30c‐1 WT and G‐to‐A mutant, supporting our model that SRSF3 binding to CNNC sites may disrupt double‐stranded RNA regions (Jones *et al*, 2022). Whether SRSF3 binding leads to changes in the pri‐miR‐30c‐1 structure or the structure of other pri‐miRNAs to mediate their efficient processing by the Microprocessor remains to be determined.

Polycistronic pri‐miRNAs pose a challenge for the Microprocessor complex compared with single pri‐miRNAs as the long polycistrons can fold into complex structures. miR‐17‐92 adopts a folded globular structure *in vitro* where some Drosha cleavage sites are buried inaccessible to the Microprocessor (Chaulk *et al*, 2011). In support of this, the processing of the six miRNAs of the cluster has been shown to occur hierarchically with some sites being preferentially processed (Donayo *et al*, 2019). Based on *in vitro* miR‐17‐92 structure model, Chakraborty & Krishnan (2017) proposed that a *trans*‐acting factor is required to unfold miR‐17‐92 structure to provide access to Drosha to the basal junctions (Chakraborty & Krishnan, 2017). Our in‐cell SHAPE‐MaP data suggest that SRSF3 could serve as this *trans*‐acting factor that disrupts base‐paired regions and through RS‐domain interactions between CNNC sites may shape not only the local base pairing interactions but also the global miR‐17‐92 structure, favouring the processing of miR‐17 and miR‐20. How the SRSF3‐mediated miR‐17‐92 structure may dock Drosha for efficient cleavage at distinct basal junctions remains to be determined. Thus, we cannot exclude the possibility that SRSF3 regulates the processing of miR‐17‐92 cluster through mechanisms beyond the modulation of its RNA structure. Our iCLIP data demonstrated that SRSF3 binds to many polycistronic miRNAs (Ratnadiwakara *et al*, 2018). Further investigations are required to uncover whether SRSF3 plays a broader role in rearranging polycistronic miRNAs and the atomic details of SRSF3‐miRNA interactions within miRNA clusters.

The preferential SRSF3‐mediated processing of the paralogs miR‐17 and miR‐20a led to enhanced cell cycle progression and proliferation in both mouse pluripotent and human cancer cells through the regulation of the cell cycle inhibitor CDKN1A/p21. Functional studies have suggested that the biological effects of miR‐17‐92 heavily depend on the cellular context (Olive *et al*, 2013). miR‐17‐92 has been implicated as both an oncogene and tumour suppressor depending on the relative abundance of individual miR‐17‐92 miRNAs (He *et al*, 2005; Zhang *et al*, 2009). Our data linking SRSF3 binding to miR‐17‐92 to mediate structural changes in the polycistron provide a mechanism by which relative levels of the six miR‐17‐92 miRNAs could be controlled. Importantly, our CRC patient data demonstrate that post‐transcriptional regulation of miR‐17‐92 by SRSF3 plays a central role in tumorigenesis and RBPs such as SRSF3 can define the functional output of miRNA clusters. The SRSF3‐miR‐17/20a‐CDKN1A “signature” was sufficient to classify patients with poorly differentiated tumours. While we do not have the survival information of the patients used for this study, the high‐grade or poorly differentiated histology is known to be associated with poor patient survival (Blenkinsopp *et al*, 1981; Jass *et al*, 1986; Compton, 1999). As the G‐to‐A mutation within the terminal loop of pri‐mir‐30c‐1 is associated with breast and gastric cancer (Fernandez *et al*, 2017) and a C‐to‐T mutation within the CNNC downstream of pri‐miR‐16 has been associated with chronic lymphocytic leukaemia (Calin *et al*, 2005), SRSF3‐mediated pri‐miRNA processing may play a key role in tumorigenesis (Fig 6H), suggesting that the concurrent dysregulation of SRSF3 and oncogenic miRNAs may be one of the hallmark features of cancer cells and warranting a further investigation on the role of RBPs modulating miRNA structures in cancer pathogenesis.

## Materials and Methods

### Reagents

RQ1 RNase‐free DNaseI (Promega, #M6101), SuperScript III First strand‐synthesis system (Thermo Fisher Scientific, #18080051), RNase A (Merck, #10109242001), anti‐EGFP (Abcam, UK, #ab290), anti‐SRSF3 (Merck, #WH0006428M8), anti‐CDKN1A (Cell Signalling Technologies, #2947), anti‐GAPDH (Cell Signalling Technologies, #2118S), anti‐TP53 (Cell Signalling Technologies, #2524), anti‐HA tag (ThermoFisher Scientific, #MA5‐25644), TaqMan® MicroRNA Reverse Transcription Kit (ThermoFisher Scientific, #4366596), SensiFAST Probe Hi‐ROX Kit (Bioline, #BIO‐82005), Pierce Firefly Luciferase Glow Assay Kit (ThermoFisher Scientific, #16176), Dynabeads Protein G beads (Thermo Fisher Scientific, #10009D), TRI Reagent (Sigma‐Aldrich, #T9424), Luminaris HiGreen qPCR Master Mix‐low ROX (ThermoFisher Scientific, #K0974), predesigned TaqMan probes (ThermoFisher Scientific, #4427975). NuPAGE 4–12% gradient Bis‐Tris gels (ThermoFisher Scientific, #NP0322BOX), ECL Western Blotting Detection Reagents (MERCK, #GERPN2209), Silencer Select predesigned siRNA (ThermoFisher Scientific, #4392420, SRSF3 assay ID s12732 and Negative Control No. 1), 2‐methylnicotinic acid imidazolide (MERCK, #913839), eBioscience™ Cell Proliferation Dye eFluor™ 670 (ThermoFisher Scientific, #65‐0840‐85) and cOmplete™ Mini EDTA‐free (Merck, #11836170001).

### Cell lines

The previously generated *Srsf3‐BAC* and *Srsf3* knockout iPS/ES cell lines were cultured and reprogramming performed as described (Ratnadiwakara *et al*, 2018). LIM1215 colorectal cancer cells stably overexpressing SRSF3 (SRSF3‐T2A‐EGFP) were generated by lentiviral transduction of a pCCL vector construct carrying human *SRSF3* followed by T2A self‐cleaving peptide and EGFP reporter driven by human PKG promoter. Cells stably expressing only EGFP were used as a control. Constructs carrying miR‐17‐92 WT, 17/20a‐ΔCNNC and total‐ΔCNNC DNA sequences (IDT) under the EF1alpha promoter were used for transient overexpression in wild‐type HEK293 and in HEK293 cells co‐transfected with EF1alpha promoter driven SRSF3‐GFP or GFP‐only overexpression vectors, or Silencer Select predesigned siRNA for SRSF3 or negative control (ThermoFisher Scientific). pCGN constructs with a CMV‐promoter driven HA‐tagged full‐length human SRSF3 (SRSF3‐FL) or SRSF3 without the RS‐domain (SRSF3‐ΔRS) were used for transient overexpression in LIM1215 cells.

### RNA extraction and quantitative real‐time PCR

Total RNA was isolated using the TRI Reagent (Sigma‐Aldrich) according to the manufacturer's protocol and subjected to DNaseI treatment (Promega). Total RNA was used for cDNA synthesis with SuperScript III Reverse Transcriptase (ThermoFisher Scientific). Quantitative PCR was performed in QuantStudio 6 PCR system (ThermoFisher Scientific) with Luminaris HiGreen qPCR Master Mix‐low ROX (Thermo Fisher Scientific) and 0.3 μM primer mix. miRNA expression was measured with TaqMan® MicroRNA Reverse Transcription Kit (Applied Biosystems) and SensiFAST Probe Hi‐ROX Kit (Bioline) using predesigned TaqMan probes (ThermoFisher Scientific). The fold changes in expression were calculated by the ΔΔCT method using snoRNA202 and U6 RNA as reference small RNAs in mouse and human cells, respectively. See Appendix Table S1 for primer sequences.

### Western blotting

Cells were lysed in RIPA buffer (50 mM Tris–HCl pH8, 150 mM NaCl, 1% Igepal, 0.5% sodium deoxycholate, 0.1% sodium dodecyl sulphate) with Complete protease inhibitors (Merck) and 25 μg protein was loaded on NuPAGE 4–12% gradient Bis‐Tris gels (ThermoFisher Scientific), followed by transfer to nitrocellulose membrane and antibody detection (buffers and antibodies described in Appendix Table S2). The blots were developed using ECL Western Blotting Detection Reagents (GE Healthcare) and visualised with the Biorad ChemiDoc MP Imaging System.

### RNA immunoprecipitation

LIM1215 SRSF3‐EGFP and LIM1215 EGFP control cell pellets (1 × 10^6^ cells) were suspended into NET‐2 buffer (50 mM Tris–HCl pH 7.5, 150 mM NaCl and 0.05% Igepal). The lysates were sonicated using Bioruptor (Diagenode, NJ, USA) and the supernatants used for RNA immunoprecipitation with anti‐EGFP (Abcam, UK, ChIP grade) coupled Dynabeads Protein G (ThermoFisher Scientific). The beads were washed with NET‐2 buffer. RNA was isolated from the beads and input samples (5% of the lysate) for RT–qPCR.

### Cell proliferation and cell cycle assay

LIM1215 SRSF3‐T2A‐EGFP or GFP‐only cells were stained with the cell proliferation dye eFluor670 (10 μM, ThermoFisher Scientific), and the cells were analysed by flow cytometry after 72 h. DAPI was used as a viability marker. Cell cycle distribution of ethanol fixed, RNase (100 μg/ml, Merck) and propidium iodide (50 μg/ml) treated LIM1215 cells was determined by flow cytometry.

### Luciferase assay

LIM1215 SRSF3‐T2A‐EGFP or GFP‐only expressing cells were transfected with luciferase constructs pGL‐p21UTR WT, M1, M1 and M1 + M2 (a gift from Robert Blelloch). After 48 h, the cells were analysed in triplicate using the Pierce Firefly Luciferase Glow Assay Kit (ThermoFisher Scientific).

### Patient samples

RNA from tumour and their paired normal tissues of 25 CRC patients were received from the Cabrini Hospital, Malvern, Australia. The study was approved by the Cabrini Research Governance Office (Reference #05‐11‐04‐11). All patients provided a written informed consent.

### In‐Cell SHAPE‐MaP

HEK293 cells were first transfected with Silencer Select predesigned siRNA for SRSF3 or negative control (ThermoFisher Scientific) according to the manufacturer's instructions, followed by a transfection with miR‐17‐92 WT, 17/20a‐ΔCNNC and total‐ΔCNNC expression constructs. In‐Cell SHAPE‐MaP was performed as described in (Smola & Weeks, 2018). 2‐methylnicotinic acid imidazolide (NAI, 100 μM, Merck) was used as the electrophile. NAI was quenched with DTT to achieve accurate RNA structure probing in the optimal reaction time frame. The sequencing libraries were prepared as described in (Smola & Weeks, 2018). The reverse transcription and PCR primers used for the library preparation are listed in Appendix Table S1. Sequencing was performed using Illumina MiSeq. The Shape‐MaP data were processed using ShapeMapper 2.1.5 (https://github.com/Weeks‐UNC/shapemapper2). FASTQ files from sequencing runs were directly input into ShapeMapper for read alignment to miR‐17‐92 amplicon and mutation counting. The SHAPE reactivities of the control and SRSF3 knockdown samples were compared using the ΔSHAPE (https://github.com/Weeks‐UNC/deltaSHAPE; Smola *et al*, 2015a). SuperFold (https://github.com/Weeks‐UNC/Superfold) (Smola *et al*, 2015b) was used to model the secondary structure of RNA, and the RNA secondary structure was visualised using VARNA (Darty *et al*, 2009). The bases were coloured based on their SHAPE reactivity values: (i) 0–0.4 (green), (ii) 0.4–0.85 (yellow) and (iii) 0.85 and above (red). The masked nucleotide bases (first 20 nucleotides from the 5′ and 3′) were coloured in blue.

### Statistical analysis

Two‐tailed unpaired Student's *t*‐test was performed when comparing one variable between two conditions, and one‐way ANOVA when comparing three or more independent variables. Two‐way ANOVA was performed when comparing two different categorical independent variables. The number of replicates (*n*) in all figures describes biological replicates. Linear regression analysis was performed to determine the association between gene expression and patient characteristics. All the analyses were performed using Graph Pad Prism version 9.

## Author contributions

**Madara Ratnadiwakara:** Conceptualization; formal analysis; investigation; visualization; methodology; writing – original draft; writing – review and editing. **Mohamed NM Bahrudeen:** Data curation; software; formal analysis; investigation; visualization; methodology; writing – review and editing. **Erika Aikio:** Data curation; formal analysis; validation; investigation; methodology; writing – review and editing. **Piia Takabe:** Investigation; visualization; methodology; writing – review and editing. **Rebekah M Engel:** Formal analysis; investigation. **Zileena Zahir:** Validation; investigation. **Thierry Jardé:** Formal analysis; investigation. **Paul J McMurrick:** Resources; supervision; funding acquisition. **Helen E Abud:** Resources; supervision; funding acquisition. **Minna‐Liisa Änkö:** Conceptualization; resources; data curation; formal analysis; supervision; funding acquisition; investigation; visualization; methodology; writing – original draft; project administration; writing – review and editing.

## Disclosure and competing interests statement

The authors declare that they have no conflict of interest.

## Supporting information



Appendix S1

Expanded View Figures PDF

PDF+

## Data Availability

The SHAPE‐MaP data are deposited in the Sequence Read Archive (SRA) at the NCBI (BioProject PRJNA855586). The reviewers can access the data using the following link: https://www.ncbi.nlm.nih.gov/bioproject/PRJNA855586.
