# Supplementary material for: SRSF3 shapes the structure of miR‐17‐92 cluster RNA and promotes selective processing of miR‐17 and miR‐20a
Source: EMBO Rep. 2023 Jun 12;24(7):e56021. doi: 10.15252/embr.202256021 (PMC10328067; doi:10.15252/embr.202256021)
Supplement: Supplementary file 1 — Appendix S1 [file EMBR-24-e56021-s003.pdf]

APPENDIX

SRSF3 shapes the structure of miR-17-92 cluster RNA and promotes selective processing of miR-17 and miR-20a

Madara Ratnadiwakara[1,2], Mohamed NM Bahrudeen[3], Erika Aikio[3], Piia Takabe[3], Rebekah M Engel[4,5,6], Zileena Zahir[1,2], Thierry Jardé[1,4,5], Paul J McMurrick[6], Helen E Abud[4,5] and Minna-Liisa Änkö[1,2,3*]

[1]Hudson Institute of Medical Research, Clayton 3168, Victoria, Australia; [2]School of Clinical Sciences, Department of Molecular and Translational Science, Monash University, Clayton 3800, Victoria, Australia; [3]Faculty of Medicine and Health Technology, Tampere University, Tampere 33520, Finland, [4]Department of Anatomy and Developmental Biology, Monash University, Clayton 3800, Victoria, Australia, [5]Development and Stem Cells Program, Monash Biomedicine Discovery Institute, Clayton 3800, Victoria, Australia; [6]Cabrini Monash University Department of Surgery, Cabrini Health, Malvern 3144, Victoria, Australia

*To whom correspondence should be addressed. Tel: +358 50 479 4852; Email: minna-liisa.anko@tuni.fi

TABLE OF CONTENTS

Appendix Figures and Legends

Appendix Figure S1. SRSF3 knockdown does not affect processing of the miR-17-92 17/20a-ΔCNNC or Total-ΔCNNC mutants.

Appendix Figure S2. In silico predicted structures of WT, 17/20a-ΔCNNC and Total-ΔCNNC miR-17-92.

Appendix Figure S3. Arc plots and secondary structures predicted based on SHAPE reactivities for 17/20a-ΔCNNC and total-ΔCNNC miR-17-92.

Appendix Tables and Legends

Appendix Table S1. Primers used in this study.

Appendix Table S2. Buffers and antibodies used in this study.

Appendix Table S3. QC summary of SHAPE-MaP analysis.

Appendix Table S4. Expression of miR-17/20a targets associated with cell cycle regulation.

Appendix Table S5. Expression of *SRSF3*, miR-17/20a and *CDKN1A* in CRC tumours compared to their paired normal samples.

APPENDIX FIGURES AND LEGENDS

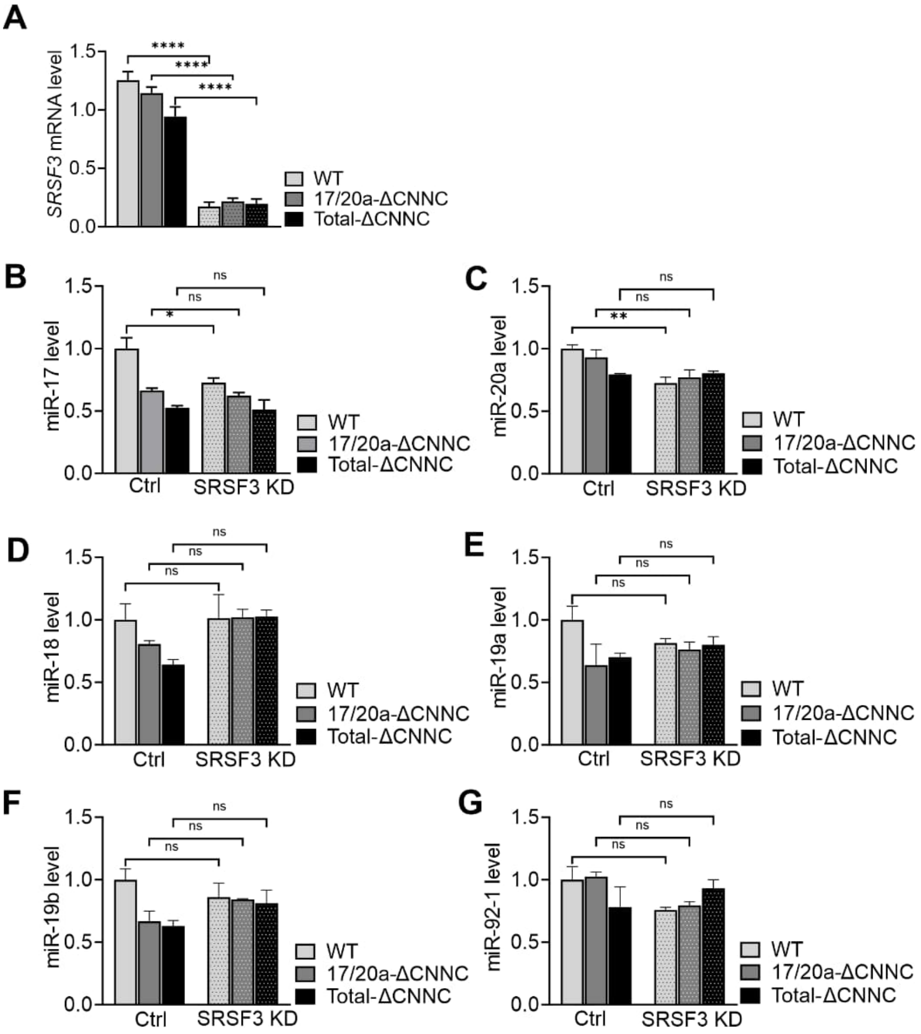

Appendix Figure S1 - SRSF3 knockdown does not affect processing of the miR-17-92 17/20a-ΔCNNC or Total-ΔCNNC mutants.

A RT-qPCR analysis of SRSF3 expression in SRSF3 KD or control (Ctrl) HEK293 expressing WT, 17/20a-ΔCNNC or total-ΔCNNC miR17-92 construct (data as mean ± SEM, n=4, biological replicates).

B-G TaqMan analysis of miR-17-92 miRNAs in SRSF3 depleted (SRSF3 KD) or control (Ctrl) HEK293 cells overexpressing SRSF3-GFP or GFP control together with WT, 17/20a-ΔCNNC or total-ΔCNNC miR17-92 (*p<0.05, **p<0.01, ***p<0.001, ****p<0.0001, #all samples significantly different compared to ctrl, One-Way ANOVA, data as mean ± SEM, n=4, biological replicates).

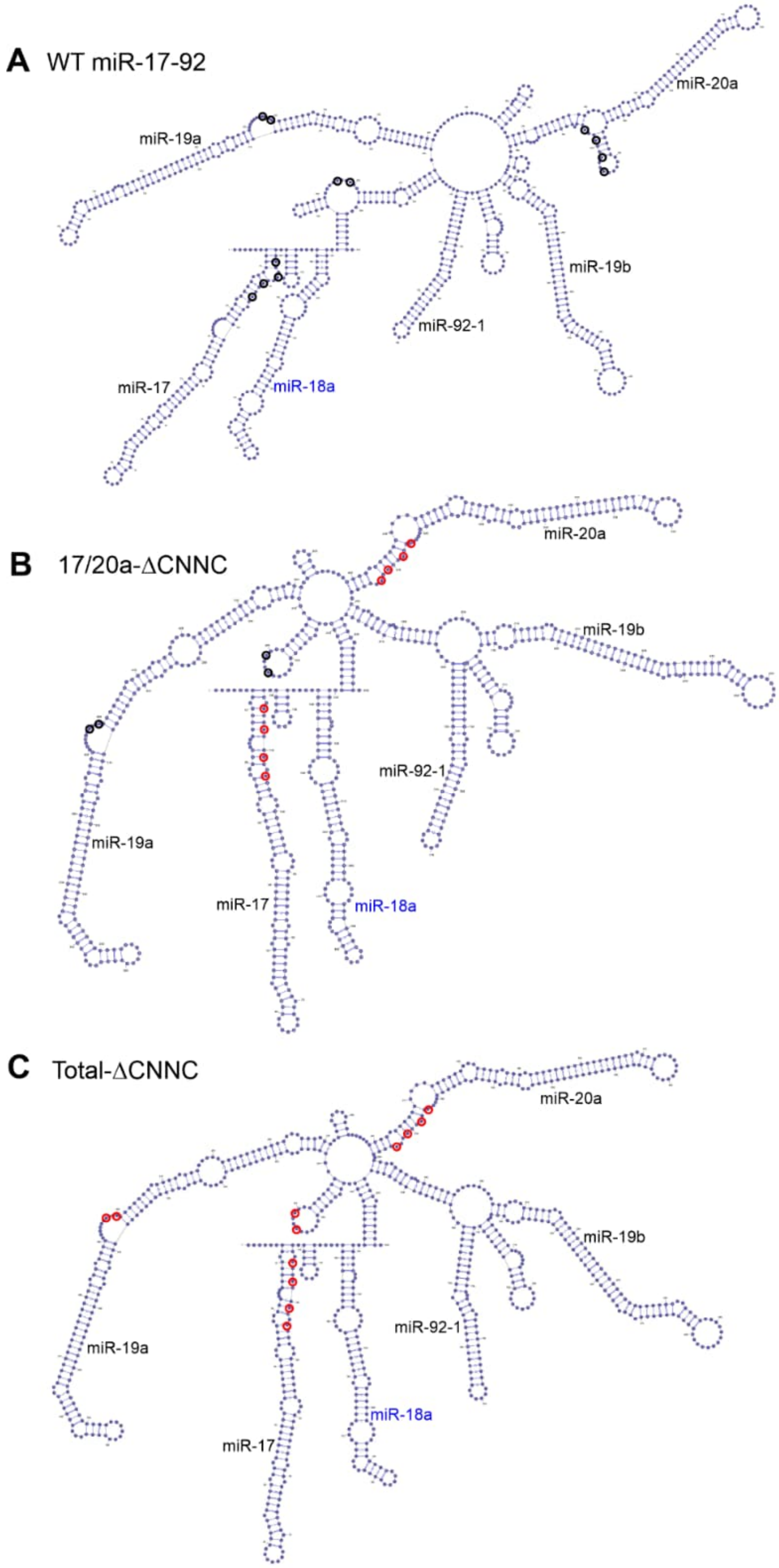

Appendix Figure S2 - *In silico* predicted structures of WT, 17/20a-ΔCNNC and Total-ΔCNNC miR-17-92.

A-C *In silico* minimum free energy RNA structure predictions of WT (A), 17/20a-ΔCNNC (B), and total-ΔCNNC (C) miR-17-92 sequences using SuperFold. SHAPE reactivity data was not

used for these predictions. The miR-18a stem loop (marked in blue) was not thermodynamically favourable and not correctly formed in the minimum free energy structure. The mutated nucleotides within the CNNC sites are circled in red, and the corresponding WT nucleotides circles in black.

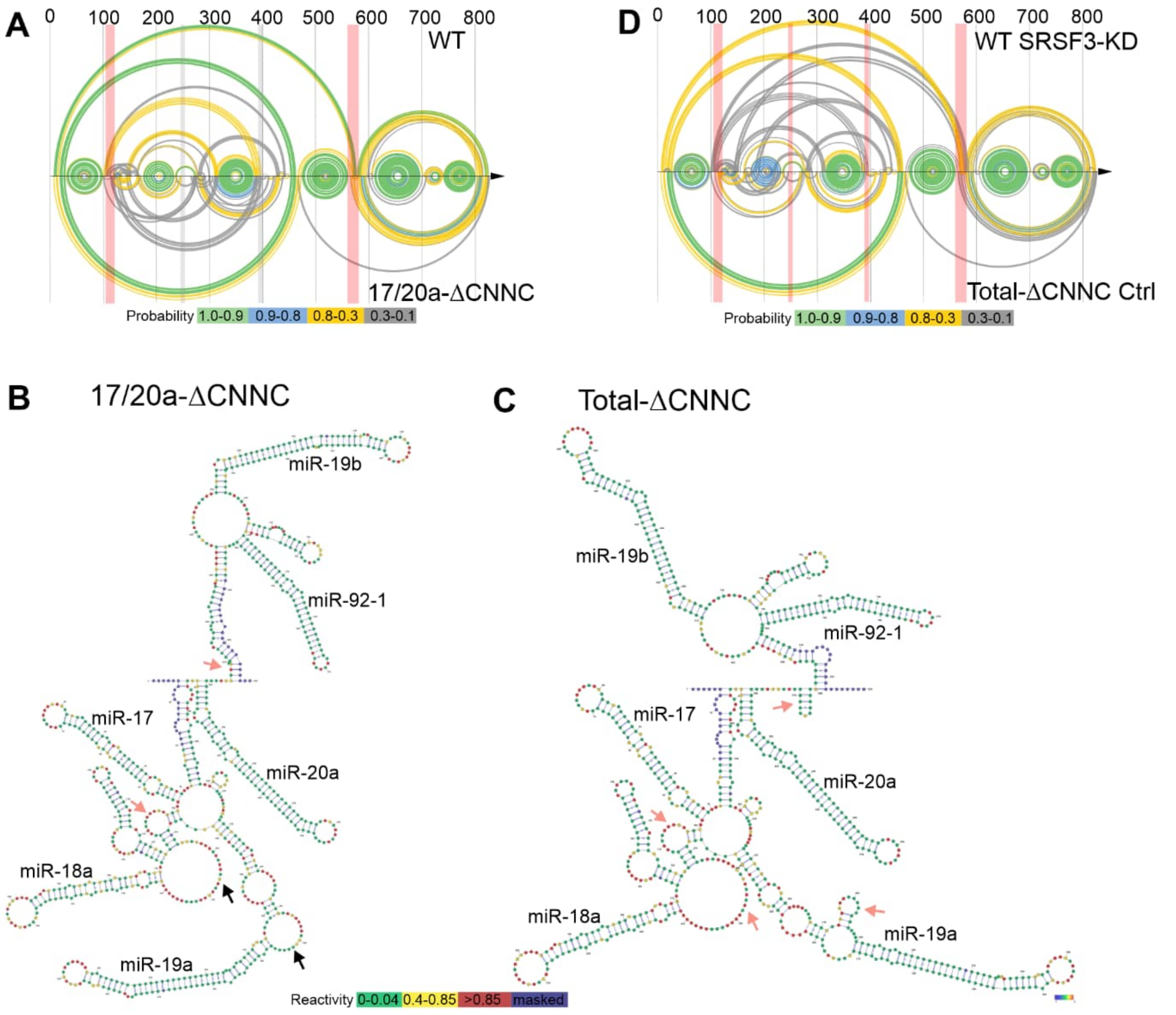

Appendix Figure S3 - Arc plots and secondary structures predicted based on SHAPE reactivities for 17/20a-ΔCNNC and total-ΔCNNC miR-17-92.

A Arc plot depicting base pairing probabilities within WT and 17/20a-ΔCNNC miR-17-92 in control cells.

B, C Secondary structure prediction of 17/20a-ΔCNNC (B) and total-ΔCNNC (C) miR-17-92 in control cells based on SHAPE reactivities.

D Arc plot depicting base pairing probabilities within WT miR-17-92 in SRSF3-KD cells and total-ΔCNNC miR-17-92 in control cells.

APPENDIX TABLES AND LEGENDS

Appendix Table S1 - Primers used in this study (F= forward primer, R= reverse primer).

Gene	Sequence	
Srsf3 (mouse)	F	TGAATTAGAACGGGCTTTTGG
	R	TTCACCATTCGACAGTTCCAC
Hprt (mouse)	F	TGTTGTTGGATATGC
	R	TGCGCTCATCTTAGG
Pri-miRNA 92-1 (mouse)	F	TCTGCTGTGCAAATCCATGC
	R	TCTTCTGGTCACAATCCCCAC
Pri-miRNA 20a (mouse)	F	CAAAACTGATGGTGGCCTGC
	R	GCTCAATAACAGGACAGTTGGC
Cdkn1a/p21 (mouse)	F	TTGCACTCTGGTGTCTGAGC
	R	TGCGCTTGGAGTGATAGAAA
Trp53/p53 (mouse)	F	CCCGAGTATCTGGAAGACAGG
	R	GTAAGGATAGGTCGGCGGTTC
Drosha (mouse)	F	GAAGTCACCGTGGAGCTGAGTA
	R	ATCATTGCATGCTGACAGACATC
Dicer (mouse)	F	CAGCTCTGGACCATAACACAATTG
	R	AGGTCGCCCCTGATCTGAT
Dgcr8 (mouse)	F	TCAAGGTCCGCCCTGTTTAT
	R	GAGGCACCAAAAGGCTCACTT
Xpo5 (mouse)	F	GACGCAGAACATGGAAAGAATCT
	R	TGTCTTCATTTGTTGGTACTTGTTTACA
Ago2 (mouse)	F	GCGTCAACAACATCCTGCT
	R	CTCCCAGGAAGATGACAGGT
SRSF3 (human)	F	AACGGGCTTTTGGCTACTATG
	R	TTCACCATTCGACAGTTCCAC
HPRT (human)	F	CTGAGGATTTGGAAAGGGTGT
	R	GTAATCCAGCAGGTCAGCAAA
CDKN1A/p21 (human)	F	CAGCAGAGGAAGACCATGTG
	R	CGGCGTTTGGAGTGGTAGA
TP53 (human)	F	AAGTCTGTGACTTGCACGTACTCC

	R	GTCATGTGCTGTGACTGCTTGTAG
miR-17-92 RT P1 (SHAPE)		TCTTCTGGTCACAATCCCCA
miR-17-92 RT P2 (SHAPE)		CTGTCACATCAGATAGACCAGGCAGAT TCT
miR-17-92 P1 (SHAPE)	F	GACTGGAGTTCAGACGTGTGCTCTTCCGAT CTNNNNNGCCAAGCAAGTATATAGGTG
	R	CCCTACACGACGCTCTTCCGATCTNNNNN TCTTCTGGTCACAATCCCCA
miR-17-92 P2 (SHAPE)	F	GACTGGAGTTCAGACGTGTGCTCTTCCGAT CTNNNNNGTAAAACTGAAGATTGTGAC
	R	CCCTACACGACGCTCTTCCGATCTNNNNN GATTCTACATCGACACAATA-

Appendix Table S2 - Buffers and antibodies used in this study.

Western blot running buffer	Invitrogen NuPAGE MES SDS Running Buffer (20X), Cat no.NP0002
Western blot transfer buffer	Invitrogen NuPAGE Transfer Buffer (20X), Cat no. NP00061
SRSF3 antibody	MERCK, Cat no. WH0006428M8
CDKN1A antibody	Cell Signalling Technologies, Cat no. 2947
GAPDH antibody	Cell Signalling Technologies, Cat no. 2118S
TP53 antibody	Cell Signalling Technologies, Cat no. 2524
HA tag antibody	ThermoFisher Scientific Cat no. MA5-25644

Appendix Table S3 - QC summary of SHAPE-MaP analysis. NAI=NAI-treated cells; DMSO=vehicle treated cells; DCC=denaturated control RNA; Ctrl=control siRNA; KD=SRSF3 siRNA (knockdown).

		WT miR-17-92		total-ΔCNNC		17/20a-ΔCNNC	
QC Parameter		Ctrl	KD	Ctrl	KD	Ctrl	KD
Overall read alignment rate	NAI	92.3%	80.2%	98.1%	97.9%	97.9%	97.8%
	DMSO	94.5%	80.7%	97.8%	98.2%	98.4%	97.8%
	DCC	98.5%	98.5%	97.3%	97.3%	96.1%	96.1%
Read depth check: % of nucleotides meet the minimum read depth of 1000		100%	100%	100%	100%	100%	100%
Mutation rate check: % of nucleotides have positive mutation rate above background		80.8%	90.9%	92.9%	88.6%	94.3%	93.0%
High background check: % of nucleotides have high background mutation rates		0.1%	0%	0.6%	0.6%	0.8%	0.6%
Highly reactive check: % of nucleotides show high apparent reactivity		9.1%	8.0%	17.4%	16.5%	15.9%	14.2%

Appendix Table S4 - Expression of miR-17/20a targets associated with cell cycle regulation. The RNA-sequencing data used for the analysis is based on (Ratnadiwakara et al, 2018). Ctrl=control and KO=SRSF3-knockout.

Gene ID	Gene Name	Fold-change (Ctrl vs KO)
ENSMUSG00000070348	*Ccnd1*	0.85
ENSMUSG00000000184	*Ccnd2*	1.42
**ENSMUSG00000023067**	***Cdkn1a***	**1.65**
ENSMUSG00000027490	*E2f1*	0.89
ENSMUSG00000022105	*Rb1*	1.11
ENSMUSG00000027641	*Rbl1*	0.94
ENSMUSG00000057329	*Bcl2*	1.49

ENSMUSG00000052934	*Fbxo31*	0.95
ENSMUSG00000020184	*Mdm2*	1.34
ENSMUSG00000034462	*Pkd2*	1.19
ENSMUSG00000013663	*Pten*	1.01
ENSMUSG00000020167	*Tcf3*	0.84
ENSMUSG00000024515	*Smad4*	1.10
ENSMUSG00000031016	*Wee1*	0.79
ENSMUSG00000022346	*Myc*	0.93
ENSMUSG00000031666	*Rbl2*	1.33

Appendix Table S5 - Expression of *SRSF3*, miR-17/20a and *CDKN1A* in CRC tumours compared to their paired normal samples. The red arrows indicate patients with *SRSF3*-miR-17/20a-*CDKN1A* signature, ↑ indicates increased and ↓ indicates decreased expression relative to paired normal.

Patient #	*SRSF3*	*CDKN1A*	miR-17	miR-20a
1	↑	↓	↑	↑
2	↑	↓	↑	↑
3	↓	↓	↓	↑
4	↑	↓	↑	↑
5	↑	↓	↑	↑
6	↓	↑	↑	↑
7	↑	↓	↑	↑
8	↑	↓	↑	↑
9	↑	↓	↑	↑
10	↑	↓	↑	↑
11	↑	↓	↑	↑
12	↓	↓	↑	↑
13	↓	↓	↓	↑
14	↑	↑	↑	↑
15	↑	↓	↑	↑
16	↑	↓	↑	↑
17	↑	↓	↑	↑
18	↓	↓	↑	↑

19	↑	↓	↑	↑
20	↓	↑	↑	↓
21	↓	↓	↓	↓
22	↑	↑	↓	↓
23	↑	↑	↑	↑
24	↑	↓	↓	↑
25	↑	↓	↑	↓
