# Supplementary material for: SRSF3 shapes the structure of miR‐17‐92 cluster RNA and promotes selective processing of miR‐17 and miR‐20a
Source: EMBO Rep. 2023 Jun 12;24(7):e56021. doi: 10.15252/embr.202256021 (PMC10328067; doi:10.15252/embr.202256021)
Supplement: Supplementary file 2 — Expanded View Figures PDF [file EMBR-24-e56021-s002.pdf]

Expanded View Figures

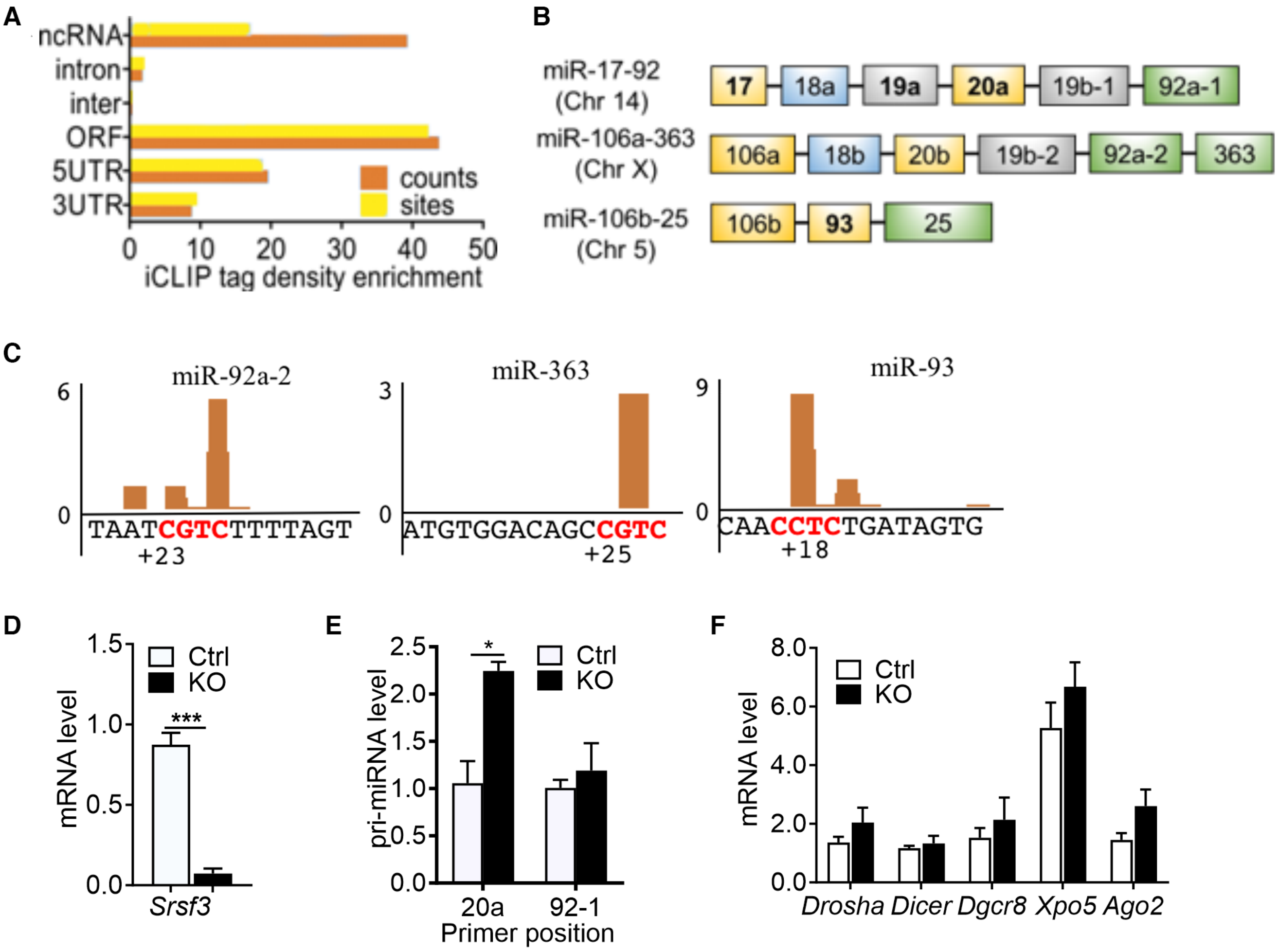

Figure EV1. SRSF3 binds to miR-17-92 miRNA and its paralog clusters and affects the mature miRNA levels in pluripotent stem cells.

A Distribution of significant SRSF3 crosslink sites (FDR < 0.05) over different transcript regions normalised to feature length. Data from (Ratnadiwakara *et al*, 2018).

B Schematic illustration of three paralog clusters miR-17-92, miR-106a-363 and miR-106b-25. The colours mark miRNAs of the same family. MicroRNAs bound by SRSF3 at 16–18 nt downstream of the Drosha cleavage site based on data in Ratnadiwakara *et al* (2018) are marked in bold.

C SRSF3 iCLIP binding peaks within the components of paralogue clusters miR-106a-363 and miR-106b-25 in mouse ES cells. The CNNC motifs are marked in red colour. The 3p position is counted from the 3′ Drosha cleavage site as described in (Auyeung *et al*, 2013), iCLIP data from (Ratnadiwakara *et al*, 2018).

D RT–qPCR quantification of *Srsf3* expression in *Srsf3*-knockout (KO) and control (Ctrl) iPSCs (***$P < 0.001$, two-tailed unpaired Student's *t*-test, data as mean ± SEM, $n = 3$, biological replicates).

E RT–qPCR quantification of pri-miRNA expression in *Srsf3*-KO (KO) and control (Ctrl) iPSCs. Two different primer pairs around miR-20a and miR-92-1 stem-loop regions were used (*$P < 0.05$, two-tailed unpaired Student's *t*-test, data as mean ± SEM, $n = 3$, biological replicates).

F RT–qPCR quantification of *Drosha*, *Dicer*, *Dgcr8*, *Xpo5* and *Ago2* mRNA levels in *Srsf3*-KO (KO) and control (Ctrl) iPSCs ($P > 0.05$ for all genes analysed, two-tailed unpaired Student's *t*-test, data as mean ± SEM, $n = 3$, biological replicates).

© 2023 The Authors

Figure EV2. SRSF3 binding to the CNNC sites is essential for miR-17-92 processing.

A RT–qPCR analysis of wild-type (WT) miR17-92, 17/20a-ΔCNNC and total-ΔCNNC construct expression in HEK293T cells (data as mean ± SEM, *n* = 4, biological replicates).

B RT–qPCR analysis of wild-type (WT) miR17-92, 17/20a-ΔCNNC and total-ΔCNNC construct expression in HEK293T cells overexpressing SRSF3-GFP or GFP control (data as mean ± SEM, *n* = 4, biological replicates).

C–H TaqMan analysis of miR-17-92 miRNAs in HEK293 cells overexpressing SRSF3-GFP or GFP together with WT, 17/20a-ΔCNNC or total-ΔCNNC miR17-92 (*$P < 0.05$, **$P < 0.01$, ***$P < 0.001$, ****$P < 0.0001$, #all samples significantly different compared with control (Ctrl), One-Way ANOVA, data as mean ± SEM, *n* = 4, biological replicates).

I Co-Immunoprecipitation in SRSF3-GFP or GFP-only expressing LIM1215 cells. In = input, IP = immunoprecipitation. The immunoprecipitation was performed with an anti-GFP antibody and the Western blot detection with an anti-SRSF3 antibody.

 © 2023 The Authors

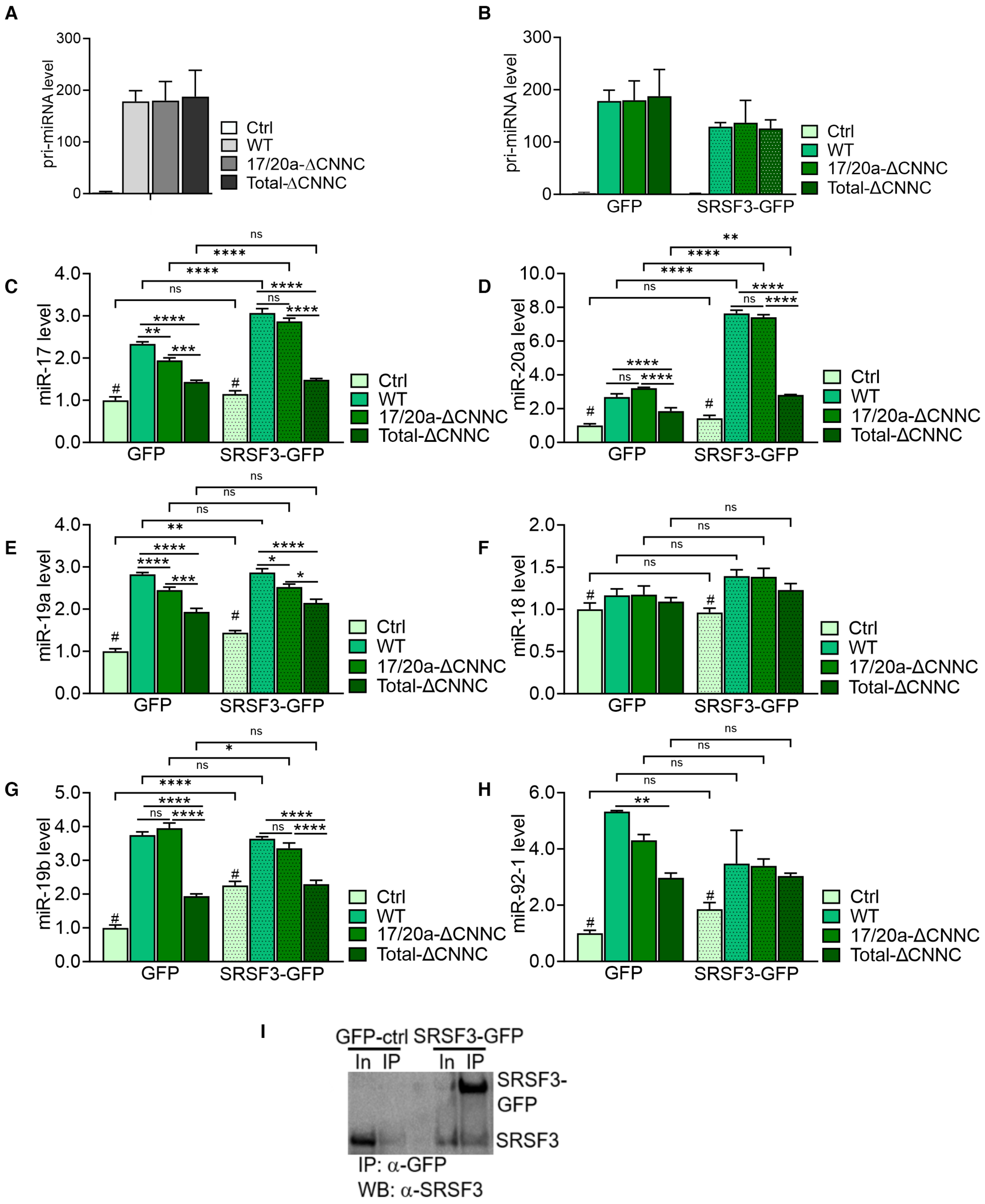
A
B
C
D
E
F
G
H
I
pri-miRNA level
Ctrl
WT
17/20a-ΔCNNC
Total-ΔCNNC
GFP
SRSF3-GFP
miR-17 level
miR-20a level
miR-19a level
miR-18 level
miR-19b level
miR-92-1 level
GFP-ctrl SRSF3-GFP
In IP In IP
SRSF3-GFP
SRSF3
IP: α-GFP
WB: α-SRSF3

Figure EV2.

Figure EV3. SRSF3 rearranges the miR-17-92 secondary structure through CNNC sites.

A WT miR-17-92 SHAPE reactivities in control (Ctrl) and SRSF3-KD cells based on ShapeMapper2. Red: SHAPE≥0.85, Yellow: 0.85 > SHAPE > 0.4 and Black: SHAPE ≤ 0.4. The bar below represents miR-17-92 transcript with the miRbase predicted miRNA active strands marked in dark blue and star strands in light blue.

B, C Comparison of 17/20a-ΔCNNC (B) and total-ΔCNNC (C) miR-17-92 SHAPE reactivities to WT miR-17-92 SHAPE reactivities. *First panel*: Mean reactivities, calculated over 11 nt sliding windows. *Second panel*: ΔSHAPE reactivity differences between WT and 17/20a-ΔCNNC or total-ΔCNNC miR-17-92. Regions with higher reactivity in WT miR-17-92 are marked with blue shading and regions with higher reactivity in 17/20a-ΔCNNC or total-ΔCNNC with grey shading. *Third panel*: The 17/20a-ΔCNNC to WT miR-17-92 or total-ΔCNNC to WT miR-17-92 reactivity ratios. The boxes mark CNNC sites (black WT, red CNNC sites mutated).

D Comparison of miR-17-92 SHAPE reactivities between 17/20a-ΔCNNC and total-ΔCNNC miR-17-92. *First panel*: Mean reactivities, calculated over 11 nt sliding windows. *Second panel*: ΔSHAPE reactivity differences between 17/20a-ΔCNNC and total-ΔCNNC miR-17-92. Regions with higher reactivity in 17/20a-ΔCNNC are marked with light grey shading and regions with higher reactivity in total-ΔCNNC miR-17-92 are marked with dark grey shading. *Third panel*: The total-ΔCNNC to 17/20a-ΔCNNC reactivity ratios. The red boxes mark the mutated CNNC sites.

E Visualisation and comparison of 17/20a-ΔCNNC miR-17-92 SHAPE reactivities in control and SRSF3-KD cells. *First panel*: Mean control (grey) and SRSF3-KD (brown) SHAPE reactivities, calculated over 11 nt sliding windows. *Second panel*: ΔSHAPE reactivity differences between control and SRSF3-KD. Regions with higher reactivity in control are marked with grey shading and regions with higher reactivity in SRSF3-KD with brown shading. *Third panel*: The SRSF3-KD to control reactivity ratios. The boxes mark CNNC sites (black WT, red CNNC sites mutated).

F Visualisation and comparison of WT miR-17-92 SHAPE reactivities in SRSF3-KD cells and total-ΔCNNC miR-17-92 SHAPE reactivities in control cells. *First panel*: Mean WT miR-17-92 SRSF3-KD (red) and total-ΔCNNC miR-17-92 control (grey) SHAPE reactivities, calculated over 11 nt sliding windows. *Second panel*: ΔSHAPE reactivity differences between WT miR-17-92 SRSF3-KD and total-ΔCNNC miR-17-92 control. Regions with higher reactivity in WT miR-17-92 SRSF3-KD are marked with red shading and regions with higher reactivity in total-ΔCNNC miR-17-92 control with grey shading. *Third panel*: The total-ΔCNNC miR-17-92 control to WT miR-17-92 SRSF3-KD reactivity ratios. The red boxes mark the mutated CNNC sites.

© 2023 The Authors

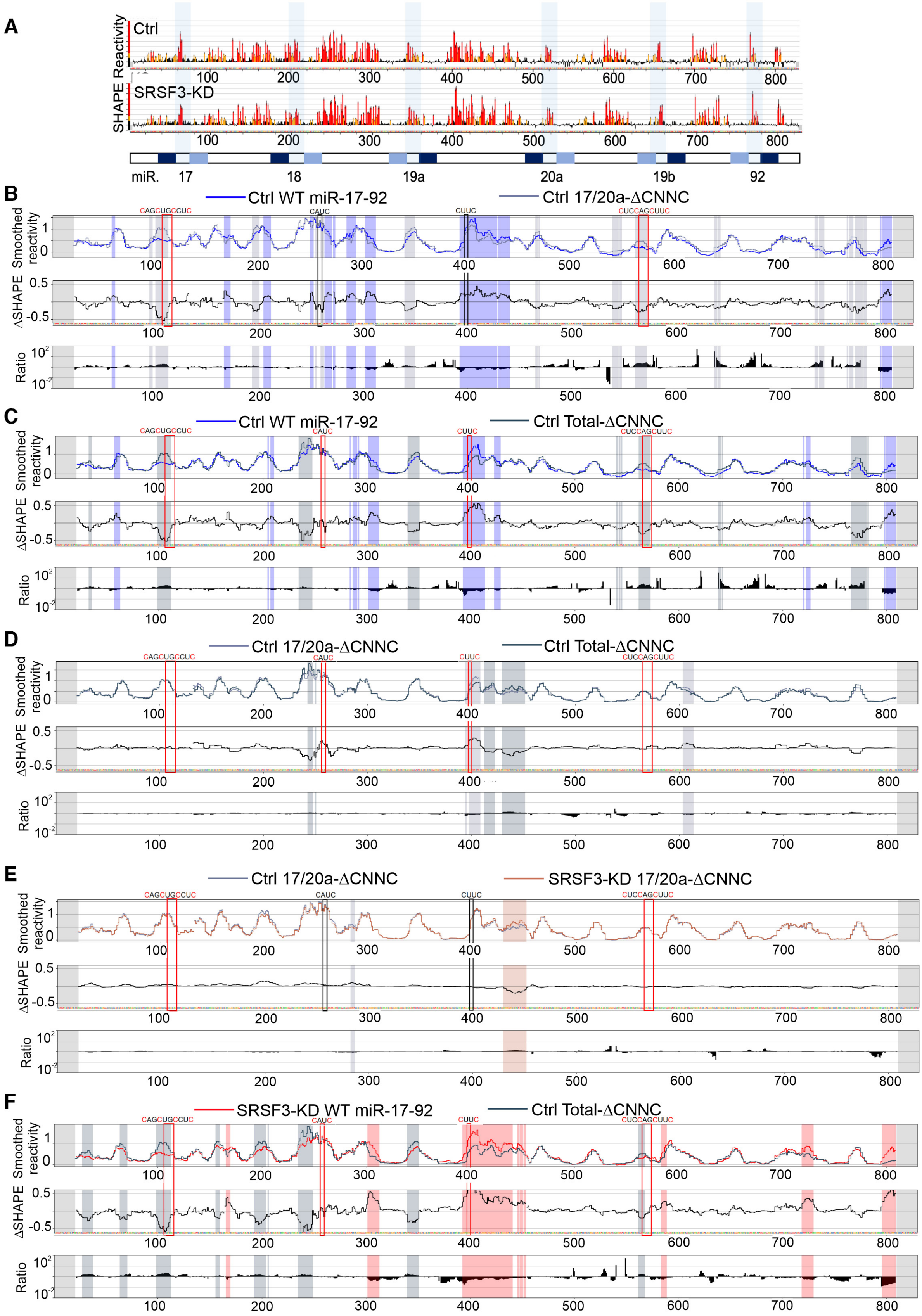
A
Ctrl
SRSF3-KD
SHAPE Reactivity
miR. 17 18 19a 20a 19b 92
B
Ctrl WT miR-17-92
Ctrl 17/20a-ΔCNNC
Smoothed reactivity
ΔSHAPE
Ratio
C
Ctrl WT miR-17-92
Ctrl Total-ΔCNNC
D
Ctrl 17/20a-ΔCNNC
Ctrl Total-ΔCNNC
E
Ctrl 17/20a-ΔCNNC
SRSF3-KD 17/20a-ΔCNNC
F
SRSF3-KD WT miR-17-92
Ctrl Total-ΔCNNC
CAGCUGCCUC
CAUC
CUUC
CUCCAGCUUC

Figure EV3.

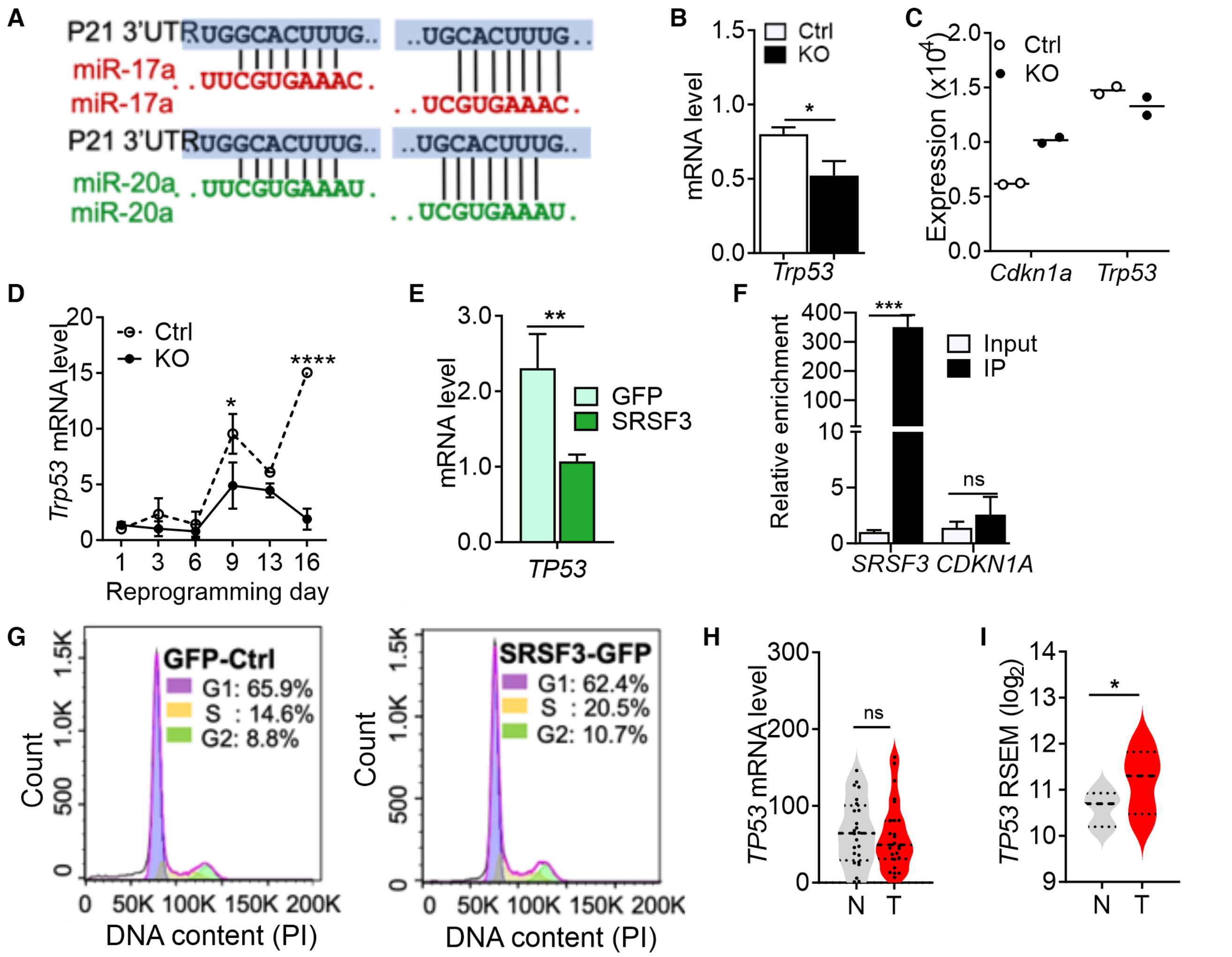

Figure EV4. SRSF3 controls CDKN1a/p21 levels through miR-17/20a independent of p53.

A Schematic of miR-17 and miR-20a seed regions that target the *Cdkn1a* 3′UTR at two sites.

B RT–qPCR quantification of *Trp53* (p53) mRNA levels in *Srsf3*-knockout (KO) and control (Ctrl) iPSCs (*$P < 0.05$, two-tailed unpaired Student's *t*-test, data as mean ± SEM, $n = 3$, biological replicates).

C Quantification of *Cdkn1a* and *Trp53* expression from *Srsf3*-KO and Ctrl RNA-sequencing data from (Ratnadiwakara *et al*, 2018; data as mean, $n = 2$, biological replicates).

D Quantification of *Trp53* mRNA expression by RT–qPCR during reprogramming in *Srsf3*-KO (KO) and control (Ctrl) cells (*$P < 0.05$, ****$P < 0.0001$, two-way ANOVA, data as mean ± SEM, $n = 3$, biological replicates).

E RT–qPCR quantification of *TP53* (p53) mRNA levels in SRSF3 overexpressing (SRSF3-GFP) and control (GFP) LIM1215 cells (**$P < 0.01$, two-tailed unpaired Student's *t*-test, data as mean ± SEM, $n = 3$, biological replicates).

F RNA immunoprecipitation (IP) of *SRSF3* (positive control) and *CDKN1A* mRNAs in GFP-only and SRSF3-GFP expressing LIM1215 cells (***$P < 0.001$, Two-tailed unpaired Student's *t*-test, data as mean ± SEM, $n = 3$, biological replicates).

G Representative histograms of propidium iodide-stained control (GFP) and SRSF3-T2A-GFP overexpressing (SRSF3-GFP) LIM1215 cells analysed by flow cytometry. Histograms were generated using cell cycle analysis tool in FlowJo software (Tree Star). Per cent cells in each phase are indicated in the inset.

H RT–qPCR quantification of *TP53* mRNA levels in colorectal tumours and their paired normal samples (ns = not significant, two-tailed unpaired Student's *t*-test, $n = 25$, biological replicates, central band = median, dotted lines = 25th and 75th percentile).

I Relative *TP53* expression in TCGA COAD data (version 2016_01_28, tumour $n = 459$ and normal $n = 41$, *$P < 0.05$, two-tailed unpaired Student's *t*-test, biological replicates, central band = median, dotted lines = 25th and 75th percentile).

© 2023 The Authors
