# Supplementary material for: SRSF3 shapes the structure of miR‐17‐92 cluster RNA and promotes selective processing of miR‐17 and miR‐20a
Source: EMBO Rep. 2023 Jun 12;24(7):e56021. doi: 10.15252/embr.202256021 (PMC10328067; doi:10.15252/embr.202256021)
Supplement: Supplementary file 4 [file EMBR-24-e56021-s004.pdf]

EMBO reports

# SRSF3 shapes the structure of miR-17-92 cluster RNA and promotes selective processing of miR-17 and miR-20a

Madara Ratnadiwakara, Mohamed Bahrudeen, Erika Aikio, Piia Takabe, Rebekah Engel, Zileena Zahir, Thierry Jardé, Paul McMurrick, Helen Abud, and Minna-Liisa Änkö
**DOI: 10.15252/embr.202256021**

*Corresponding author(s): Minna-Liisa Änkö (minna-liisa.anko@tuni.fi)*

**Review Timeline:**

| | |
|---|---|
| Submission Date: | 25th Aug 22 |
| Editorial Decision: | 29th Sep 22 |
| Revision Received: | 28th Mar 23 |
| Editorial Decision: | 21st Apr 23 |
| Revision Received: | 4th May 23 |
| Accepted: | 15th May 23 |

*Editor: Esther Schnapp*

## Transaction Report:

(Note: With the exception of the correction of typographical or spelling errors that could be a source of ambiguity, letters and reports are not edited. Depending on transfer agreements, referee reports obtained elsewhere may or may not be included in this compilation. Referee reports are anonymous unless the Referee chooses to sign their reports.)

## 1st Editorial Decision 29th Sep 2022

Dear Prof. Änkö,

Thank you for the submission of your manuscript to EMBO reports. We have now received the enclosed comments from referees 1 and 2. Referee 3 could unfortunately not sent her/his report due to a family emergency. I also paste comments from an advisor I contacted before peer-review below.

As you will see, the referees acknowledge that the findings are potentially interesting. However, they also point out that the data are preliminary, and they have several suggestions for how the study could be strengthened and improved. I think all suggestions are good and should be addressed, but please let me know in case you disagree, and we can discuss the revisions further or in more detail, also in a video chat, if you like. I do think that the data on how SRSF3 shapes the structure of the miR-17-92 cluster to promote selective processing need to be strengthened, and the advisor consulted has an interesting suggestion for how this could be done. All requested controls should also be provided.

I would thus like to invite you to revise your manuscript with the understanding that the referee concerns must be fully addressed and their suggestions taken on board. Please address all referee concerns in a complete point-by-point response. Acceptance of the manuscript will depend on a positive outcome of a second round of review. It is EMBO reports policy to allow a single round of major revision only and acceptance or rejection of the manuscript will therefore depend on the completeness of your responses included in the next, final version of the manuscript.

We realize that it is difficult to revise to a specific deadline. In the interest of protecting the conceptual advance provided by the work, we recommend a revision within 3 months (29th Dec 2022). Please discuss the revision progress ahead of this time with the editor if you require more time to complete the revisions.

IMPORTANT NOTE: we perform an initial quality control of all revised manuscripts before re-review. Your manuscript will FAIL this control and the handling will be DELAYED if the following APPLIES:
1) A data availability section providing access to data deposited in public databases is missing. If you have not deposited any data, please add a sentence to the data availability section that explains that.
2) Your manuscript contains statistics and error bars based on n=2. Please use scatter blots in these cases. No statistics should be calculated if n=2.

When submitting your revised manuscript, please carefully review the instructions that follow below. Failure to include requested items will delay the evaluation of your revision.

1) a .docx formatted version of the manuscript text (including legends for main figures, EV figures and tables). Please make sure that the changes are highlighted to be clearly visible.

2) individual production quality figure files as .eps, .tif, .jpg (one file per figure). See https://wol-prod-cdn.literatumonline.com/pb-assets/embo-site/EMBOPress_Figure_Guidelines_061115-1561436025777.pdf for more info on how to prepare your figures.

3) We replaced Supplementary Information with Expanded View (EV) Figures and Tables that are collapsible/expandable online. A maximum of 5 EV Figures can be typeset. EV Figures should be cited as 'Figure EV1, Figure EV2" etc... in the text and their respective legends should be included in the main text after the legends of regular figures.

- For the figures that you do NOT wish to display as Expanded View figures, they should be bundled together with their legends in a single PDF file called *Appendix*, which should start with a short Table of Content. Appendix figures should be referred to in the main text as: "Appendix Figure S1, Appendix Figure S2" etc. See detailed instructions regarding expanded view here: <https://www.embopress.org/page/journal/14693178/authorguide#expandedview>

- Additional Tables/Datasets should be labeled and referred to as Table EV1, Dataset EV1, etc. Legends have to be provided in a separate tab in case of .xls files. Alternatively, the legend can be supplied as a separate text file (README) and zipped together with the Table/Dataset file.

4) a .docx formatted letter INCLUDING the reviewers' reports and your detailed point-by-point responses to their comments. As part of the EMBO Press transparent editorial process, the point-by-point response is part of the Review Process File (RPF), which will be published alongside your paper.

5) a complete author checklist, which you can download from our author guidelines <https://www.embopress.org/page/journal/14693178/authorguide>. Please insert information in the checklist that is also reflected in the manuscript. The completed author checklist will also be part of the RPF.

6) Please note that all corresponding authors are required to supply an ORCID ID for their name upon submission of a revised manuscript (<https://orcid.org/>). Please find instructions on how to link your ORCID ID to your account in our manuscript

tracking system in our Author guidelines
<https://www.embopress.org/page/journal/14693178/authorguide#authorshipguidelines>

7) Before submitting your revision, primary datasets produced in this study need to be deposited in an appropriate public database (see https://www.embopress.org/page/journal/14693178/authorguide#datadeposition). Please remember to provide a reviewer password if the datasets are not yet public. The accession numbers and database should be listed in a formal "Data Availability" section placed after Materials & Method (see also https://www.embopress.org/page/journal/14693178/authorguide#datadeposition). Please note that the Data Availability Section is restricted to new primary data that are part of this study. * Note - All links should resolve to a page where the data can be accessed. *
If your study has not produced novel datasets, please mention this fact in the Data Availability Section.

8) We would also encourage you to include the source data for figure panels that show essential data. Numerical data should be provided as individual .xls or .csv files (including a tab describing the data). For blots or microscopy, uncropped images should be submitted (using a zip archive if multiple images need to be supplied for one panel). Additional information on source data and instruction on how to label the files are available at
<https://www.embopress.org/page/journal/14693178/authorguide#sourcedata>.

9) Our journal also encourages inclusion of *data citations in the reference list* to directly cite datasets that were re-used and obtained from public databases. Data citations in the article text are distinct from normal bibliographical citations and should directly link to the database records from which the data can be accessed. In the main text, data citations are formatted as follows: "Data ref: Smith et al, 2001" or "Data ref: NCBI Sequence Read Archive PRJNA342805, 2017". In the Reference list, data citations must be labeled with "[DATASET]". A data reference must provide the database name, accession number/identifiers and a resolvable link to the landing page from which the data can be accessed at the end of the reference. Further instructions are available at https://www.embopress.org/page/journal/14693178/authorguide#referencesformat

10) Regarding data quantification (see Figure Legends:
https://www.embopress.org/page/journal/14693178/authorguide#figureformat)

The following points must be specified in each figure legend:

- the name of the statistical test used to generate error bars and P values,

- the number (n) of independent experiments (please specify technical or biological replicates) underlying each data point,

- the nature of the bars and error bars (s.d., s.e.m.),

- If the data are obtained from n {less than or equal to} 2, use scatter blots showing the individual data points.

Discussion of statistical methodology can be reported in the materials and methods section, but figure legends should contain a basic description of n, P and the test applied.

- Please also include scale bars in all microscopy images.

11) The journal requires a statement specifying whether or not authors have competing interests (defined as all potential or actual interests that could be perceived to influence the presentation or interpretation of an article). In case of competing interests, this must be specified in your disclosure statement. Further information: https://www.embopress.org/competing-interests

We would also welcome the submission of cover suggestions, or motifs to be used by our Graphics Illustrator in designing a cover.

As part of the EMBO publication's Transparent Editorial Process, EMBO reports publishes online a Review Process File (RPF) to accompany accepted manuscripts. This File will be published in conjunction with your paper and will include the referee reports, your point-by-point response and all pertinent correspondence relating to the manuscript.

You are able to opt out of this by letting the editorial office know (emboreports@embo.org). If you do opt out, the Review Process File link will point to the following statement: "No Review Process File is available with this article, as the authors have chosen not to make the review process public in this case."

I look forward to seeing a revised form of your manuscript when it is ready. Please use this link to submit your revision:
https://embor.msubmit.net/cgi-bin/main.plex

Yours sincerely,

Esther Schnapp, PhD
Senior Editor
EMBO reports

Referee #1:

In this study, Ratnadiwakra and co-authors investigate the role of the SR protein family SRSF3, in the processing of a miRNA cluster, miR-17-92.

There has been evidence in the literature for a role for SRSF3 in the Microprocessor-mediated processing of miRNAs via binding to a CNNC motif in the vicinity of the Drosha cleavage site. In agreement with this, the authors analysed SRSF3 iCLIP data from their previous paper in eLife and show that SRSF3 binds to multiple CNNC motifs along the miR-17-92 cluster downstream of Drosha cleavage sites. In particular, binding of SRSF3 to miR-17 and miR-20a affected its mature miRNA levels. From these experiments, the authors concluded that SRSF3 influences the selective Microprocessor-mediated processing of miR-17 and miR-20a.

The authors went on to demonstrate that the RS domain of SRSF3 is required to promote this selective miRNA processing. Use of in-cell SHAPE-MaP revealed that SRSF3 acts to rearrange the secondary structure of the miR-17-92 cluster. The authors next show that SRSF3 controls mouse and human CDKN1a (p21) levels through increased production of miR-17 and miR-20a mature miRNAs. Finally, the authors show that the SRSF3-miR-17-92-p21 axis operates in colorectal cancer cells.

The authors put forward a model where they propose that SRSF3 shapes the structure of the miR-17-92 cluster and influences selective processing of their individual miRNAs. Mechanistically, data presented in this study suggests but does not definitely prove how SRSF3 binding leads to preferential processing of the two specific miRNAs. Nonetheless, this is an important study that furthers our knowledge on the mechanism of action of auxiliary factors for miRNA processing.

The manuscript is well-written, the quality of the data is high, and the conclusions are fully supported by the data. This study paper is suitable for publication in EMBO reports.

Specific comments

When describing the experiments on Fig 2 and S2,

'We overexpressed these constructs in HEK293T cells that do not express endogenous miR-17-92 (Supplementary Figure S2A)'.

I may be missing something here but is not clear whether HEK293T cells do not normally express the miR-17-92 cluster, or whether this has been experimentally deleted. Please clarify. Irrespective of this, Fig. 2B could/should include a control of non-transfected cells besides WT, 17-20a-ΔCNNC and total-ΔCNNC.

- On Fig.4, effects of miR-17 and miR-20a on Cdkn1a seem to operate at the level of mRNA stability. Is there any reference in the literature whether miRNAs regulating this mRNA target operate via RNA stability and not via reduced mRNA translation?

Minor

- On page 11, when describing SHAPE, authors should define NAI.

Referee #2:

In this manuscript, Ratnadiwakara et al. investigate SRSF3's role in the processing of mir-17-92 cluster. Taking advantage of SRSF3 KO mouse iPS/ES cell lines established previously, the authors show that SRSF3 binds to multiple "CNNC" motifs embedded in the pri-mir-17-92 by re-analyzing their published CLIP data. They find that SRSF3 selectively enhances the biogenesis of mir-17 and mir-20a and propose remodeling pri-miRNA-structure as the underlying mechanism. The authors also demonstrate that such a regulation has potential functional role in physiology and disease.

Although potentially interesting, these results are preliminary. The major claim as shown in the title "SRSF3 shapes the structure of mir-17-92 to control selective processing of miRNAs" is not supported by the evidence provided. Most results are suggestive with key experiments such as rescue assays missing. The functional study, while nice and convincing, adds little to what is

already known.

Major concerns:

1. Fig1C. This is the key, if not the only evidence to support the claim of "selective" regulation on mir-17/20a. However, mir-19a/b and mir-92 all show somewhat reduced expression when SRSF3 was depleted, which is consistent with their mutagenesis and overexpression study. Is that possible that "selective" reduction is an artifact due to efficient processing of mir-17/20a to begin with?
2. Fig1C. Rescue assay should be performed to show these effects are specific to SRSF3. If possible, more quantitative and trustworthy method such as northern blot should be used to quantify the changes of miRNA levels.
3. Fig2B. When all miRNAs are processed from the o/e construct with comparable efficacy, the effect of "CNNC" deletion is not specific to mir-17/20a. This argues that regulation of SRSF3 is not selective as authors proposed.
4. Fig2D, SRSF3-FL and SRSF3-dRS should be tested in a rescue assay in the SRSF3 KO cells.
5. Fig2. Manipulation of SRSF3 and its binding sites were tested separately. It will be much more convincing if they can be tested together. For example, does SFSR3 (gain of function or loss of function) have no effect on the pri-17-92 with all CNNC motifs deleted?
6. Fig3. The SHAPE analysis is nice. However, how could we be certain that the structure differences are due to specific interaction between SRSF3 and mir-17-92 transcripts? Rescue assay together with the "CNNC" mutant constructs should be tested and compared by SHAPE analysis.

Advisor's comments:

I agree that it remains unclear how SRSF3 changes the pri-miRNA structure. However, as long as the SHAPE data is clear enough to indicate the rearrangement of the secondary structure, it might be OK. It would be ideal if the authors can design a mutation(s) that drives/stablizes the rearranged secondary structure (based on the SHAPE data) and show enhanced production of miR-17/20a, but I'm not sure how feasible it is.

**Point-by-point response to the referee comments (EMBOR-2022-56021V1)**

**Referee #1:**

In this study, Ratnadiwakra and co-authors investigate the role of the SR protein family SRSF3, in the processing of a miRNA cluster, miR-17-92.

There has been evidence in the literature for a role for SRSF3 in the Microprocessor-mediated processing of miRNAs via binding to a CNNC motif in the vicinity of the Drosha cleavage site. In agreement with this, the authors analysed SRSF3 iCLIP data from their previous paper in eLife and show that SRSF3 binds to multiple CNNC motifs along the miR-17-92 cluster downstream of Drosha cleavage sites. In particular, binding of SRSF3 to miR-17 and miR-20a affected its mature miRNA levels. From these experiments, the authors concluded that SRSF3 influences the selective Microprocessor-mediated processing of miR-17 and miR-20a.

The authors went on to demonstrate that the RS domain of SRSF3 is required to promote this selective miRNA processing. Use of in-cell SHAPE-MaP revealed that SRSF3 acts to rearrange the secondary structure of the miR-17-92 cluster. The authors next show that SRSF3 controls mouse and human CDKN1a (p21) levels through increased production of miR-17 and miR-20a mature miRNAs. Finally, the authors show that the SRSF3-miR-17-92-p21 axis operates in colorectal cancer cells.

The authors put forward a model where they propose that SRSF3 shapes the structure of the miR-17-92 cluster and influences selective processing of their individual miRNAs. Mechanistically, data presented in this study suggests but does not definitely prove how SRSF3 binding leads to preferential processing of the two specific miRNAs. Nonetheless, this is an important study that furthers our knowledge on the mechanism of action of auxiliary factors for miRNA processing.

The manuscript is well-written, the quality of the data is high, and the conclusions are fully supported by the data. This study paper is suitable for publication in EMBO reports.

***Specific comments:***

**1.** When describing the experiments on Fig 2 and S2, 'We overexpressed these constructs in HEK293T cells that do not express endogenous miR-17-92 (Supplementary Figure S2A)'. I may be missing something here but is not clear whether HEK293T cells do not normally express the miR-17-92 cluster, or whether this has been experimentally deleted. Please clarify. Irrespective of this, Fig. 2B could/should include a control of non-transfected cells besides WT, 17-20a-ΔCNNC and total-ΔCNNC.

We have now included the control (Ctrl, HEK293 cells without miRNA overexpression) in Figure 2B as requested. We originally left it out for clarity because the levels of endogenous pri-miR-17-92 as well as miR-17-92-derived miRNAs in HEK293 were very low compared to the expression constructs (Figure 2B and Figure EV2A, ctrl bars barely visible in the graph). We chose HEK293 cells for the 17/20a-ΔCNNC and total-ΔCNNC experiments because they express little miR-17-92 to ensure that the contributions of miRNAs derived from the endogenous miR-17-92 were minor compared to the miRNAs derived from the overexpression constructs. The expression of WT, 17/20a-ΔCNNC and total-ΔCNNC was driven by a

moderate EF1a-promoter to avoid saturation of the processing machinery but was ~200-fold higher than the endogenous level (Figure EV2A-B). We have further clarified this in the text.

**2.** On Fig.4, effects of miR-17 and miR-20a on Cdkn1a seem to operate at the level of mRNA stability. Is there any reference in the literature whether miRNAs regulating this mRNA target operate via RNA stability and not via reduced mRNA translation?

As demonstrated in Figure EV4A, miR-17 and miR-20 seed sequences are fully complementary to *CDKN1A* 3'UTR. The main mode of action of animal miRNAs is destabilization and/or translational repression of the target mRNA (reviewed recently in Iwakawa and Tomari, *Mol Cell* 2022). The mRNA degradation of the target mRNAs occurs through deadenylation, decapping and exonucleolytic cleavage. mRNA destabilization has been shown to explain most of the steady-state repression mediated by mammalian miRNAs (Eichhorn et al., *Mol Cell* 2014; reviewed in Bartel, *Cell* 2018). Even in the case of miRNAs that predominantly cause translational repression, the translational repression eventually leads to deadenylation and degradation of the target mRNA. The degradation of *CDKN1A/Cdkn1a* mRNA because of miR-17 or miR-20a activity has been previously reported in different cellular contexts (for instance, in MLL leukemia stem cells by Wong et al, *Cancer Res* 2010; in gastric cancer by Zhou et al, *BMC Cancer* 2021; in nasopharyngeal carcinoma by Chen et al, *Cancer Med* 2016). We have now included these references in the manuscript. We demonstrated the destabilisation by measuring both *CDKN1A* mRNA and p21 protein abundance following increased in miR-17 and 20a levels (Figure 5A-C). *CDKN1A/Cdkn1a* mRNA levels were consistently altered in response to changes in miR-17/20a levels both in mouse and human cells (Figure 4B-F; Figure 6B, G).

***Minor***

**3.** On page 11, when describing SHAPE, authors should define NAI.

We defined NAI in the materials and methods when mentioned for the first time in the original manuscript but have now included the definition of NAI also in the results as requested.

**Referee #2:**

In this manuscript, Ratnadiwakara et al. investigate SRSF3's role in the processing of mir-17-92 cluster. Taking advantage of SRSF3 KO mouse iPS/ES cell lines established previously, the authors show that SRSF3 binds to multiple "CNNC" motifs embedded in the pri-mir-17-92 by re-analyzing their published CLIP data. They find that SRSF3 selectively enhances the biogenesis of mir-17 and mir-20a and propose remodeling pri-miRNA-structure as the underlying mechanism. The authors also demonstrate that such a regulation has potential functional role in physiology and disease.

Although potentially interesting, these results are preliminary. The major claim as shown in the title "SRSF3 shapes the structure of mir-17-92 to control selective processing of miRNAs" is not supported by the evidence provided. Most results are suggestive with key experiments such as rescue assays missing. The functional study, while nice and convincing, adds little to what is already known.

To further support our conclusions, we have now included rescue assays and determined *in vivo* structures of the miR-17-92 CNNC-mutants. We would like to note that while SRSF3 is a well-established proto-oncogene, the molecular mechanisms underlying its tumour promoting properties remain unclear. Similarly, while SRSF3 is known to regulate pri-miRNA processing, there is little-to-no previous data on the functional consequences of SRSF3-mediated enhanced miRNA processing. Furthermore, SRSF3 has not been previously studied in the context of clustered miRNAs. Overall, the processing of clustered miRNAs in the cellular context is poorly understood, and our work contributes to filling this knowledge gap. SRSF3 acting as a modulator of RNA structure has not been previously reported and may have broader implications for understanding how SRSF3 controls RNA metabolism in cells. As such, our study represents a significant advance in our understanding of both mechanistic and functional roles of SRSF3 in pri-miRNA processing. We have described these advances more in detail in the introduction and discussion sections of the revised manuscript.

***Major concerns:***

**1.** Fig1C. This is the key, if not the only evidence to support the claim of "selective" regulation on mir-17/20a. However, mir-19a/b and mir-92 all show somewhat reduced expression when SRSF3 was depleted, which is consistent with their mutagenesis and overexpression study. Is that possible that "selective" reduction is an artifact due to efficient processing of mir-17/20a to begin with?

The reduction in endogenous miR-19a/b or miR-92 levels was not statistically significant when measured in mouse and human cells (Figure 1C, 5B and 6C). We observed a systematic reduction/increase in the endogenous miR-17 and miR-20a levels in response to SRSF3 knockdown/overexpression in mouse and human cells (Figures 1C, 5B, 6C, 6F). All miR-17-92 miRNAs were affected when SRSF3 binding sites within miR-17-92 were mutated (Figure 2B) or when SRSF3 was overexpressed at a very high level (SRSF3-FL driven by a viral CMV promoter, >100-fold overexpression in Figure 2C-D). Moderate SRSF3 overexpression resulted in a selective increase in miR-17/20a (SRSF3 driven by a human PKG promoter, <5-fold overexpression in Figure 5B). The fold-increase of SRSF3 expression in tumour samples compared to normal tissues was comparable to the PKG-driven overexpression, demonstrating that the moderate SRSF3 overexpression reflected physiological levels (compare Figure 5B and Figure 6A & G). These data demonstrated that SRSF3 binding is required for the efficient processing of the whole miR-17-92 cluster but SRSF3 specifically enhanced the processing of miR-17/20a. The loss of selectivity in miR-17-92 regulation following SRSF3-FL overexpression was likely due to the very high level of SRSF3 overexpression. We have clarified the experimental differences in the manuscript.

We have now included additional experiments where we overexpressed SRSF3 in the context of the 17/20a-ΔCNNC and total-ΔCNNC mutants. Moderate SRSF3 overexpression selectively enhanced the processing of miR-17/20a from WT miR-17-92 construct but not the total-ΔCNNC mutant (new data in Figure EV2C-D, SRSF3 expression driven by human EF1a-promoter and resulting in expression level similar to Figure 5B). SRSF3 overexpression did not significantly affect the processing of miR-18a, miR-19a/b or miR-92-1 from the WT or total-ΔCNNC miR-17-92 construct (compare GFP vs SRSF3-GFP in Figure EV2E-H). Mutating only miR17/20a CNNC sites (17/20a-ΔCNNC) had little or no effect on the levels of miR-17-92 miRNAs (Figure EV2C-H) as demonstrated also in the original manuscript in the context of the endogenous miR-17-92 (Figure 2B). Thus, moderate SRSF3 overexpression selectively enhanced miR-17/20a processing from both the endogenous and plasmid derived WT miR-17-92 (Figure 5B and Figure EV2C-H), which fully supports our conclusions.

**2.** Fig1C. Rescue assay should be performed to show these effects are specific to SRSF3. If possible, more quantitative and trustworthy method such as northern blot should be used to quantify the changes of miRNA levels.

We have now included <u>additional experiments that combine the overexpression and knockdown of SRSF3 with the CNNC-mutations</u>. SRSF3 overexpression did not rescue and SRSF3 depletion had no additional effect on the miRNA levels in the context of the CNNC mutations (Figure EV2C-H, Appendix Figure S1A-G). These data demonstrate that <u>both SRSF3 and the CNNC sites are required for the enhanced processing of miR-17-92</u>. We have also performed additional SHAPE-MaP experiments combining the CNNC mutations with SRSF3 depletion to further demonstrate that SRSF3 acts by binding to the CNNC sites (see response to points 5 and 6).

Taqman assays are a well-validated, quantitative, sensitive, specific and widely accepted methods to quantify miRNA levels (reviewed in Siddika and Heidemann, *Front Bioeng Biotech* 2021). We carefully tested the commercially validated Taqman probes used in this study. Although Northern blots have some advantages, they are difficult to quantify and are at best semi-quantitative (Ouyang et al, *Anal Chem* 2019). As many different assays were performed, we chose to use Taqman assays for higher throughput. For the human patient data, the sample material was limiting, and Northern blots were not an option.

**3.** Fig2B. When all miRNAs are processed from the o/e construct with comparable efficacy, the effect of "CNNC" deletion is not specific to mir-17/20a. This argues that regulation of SRSF3 is not selective as authors proposed.

See response to point 1. We agree that SRSF3 is essential for the efficient processing of the whole cluster but specifically enhances miR-17/20a processing. Our new data (Figure EV2B-H and Appendix Figure S1A-G) further support this conclusion.

**4**. Fig2D, SRSF3-FL and SRSF3-dRS should be tested in a rescue assay in the SRSF3 KO cells.

The siRNA used in the manuscript (Silencer Select pre-designed siRNA, ThermoFisher Scientific, #4392420, assay ID s12732) targets the coding sequence of *SRSF3* (exons 2 and 3). We chose to use this siRNA because of its efficiency and specificity. However, we observed that this siRNA significantly inhibited both endogenous SRSF3 and plasmid derived SRSF3-FL or SRSF3-RRM (see Western blot below provided for the referees). Therefore, we were not able to perform the knockdown rescue experiment, and we do not find it reasonable to repeat all the experiments in the manuscript with a newly designed siRNA targeting only the

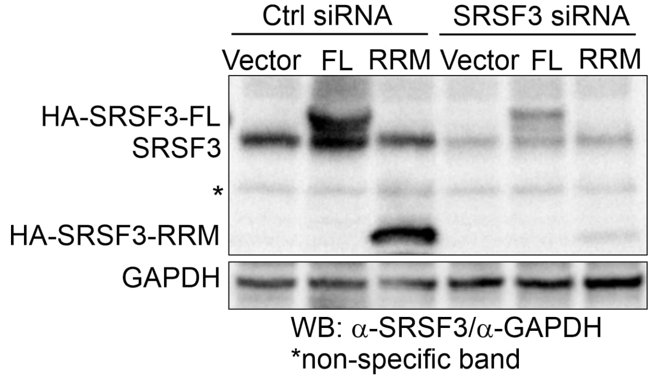


SRSF3 siRNA (assay ID s12732) efficiently silences both endogenous and plasmid-driven expression of SRSF3. GAPDH was used to control uniform sample loading.

endogenous *SRSF3* (for instance, siRNA targeting 3'UTR). However, we preformed new experiments that combined the overexpression/depletion of SRSF3 with the CNNC-mutant constructs to demonstrate that the changes in miRNA levels depended on SRSF3 binding to the CNNC sites within miR-17-92 RNA (see response to points 1 and 2). In addition, we have performed SHAPE-MaP analysis of the CNNC-mutantions in combination with SRSF3 depletion to further supports our conclusions (see response to point 5 and 6).

**5.** Fig2. Manipulation of SRSF3 and its binding sites were tested separately. It will be much more convincing if they can be tested together. For example, does SFSR3 (gain of function or loss of function) have no effect on the pri-17-92 with all CNNC motifs deleted?

We have included data combining SRSF3 overexpression/knockdown with the CNNC-mutants (Figure EV2B-H and Appendix Figure S1A-G) that fully support our conclusions. SRSF3 overexpression did not rescue the CNNC mutations and SRSF3 depletion had no effect on total-ΔCNNC miR-17-92 processing. See also responses to points 1-3.

**6.** Fig3. The SHAPE analysis is nice. However, how could we be certain that the structure differences are due to specific interaction between SRSF3 and mir-17-92 transcripts? Rescue assay together with the "CNNC" mutant constructs should be tested and compared by SHAPE analysis.

We have performed additional extensive SHAPE-MaP experiments that combined SRSF3 knockdown with the CNNC-mutations (Figure 3G-J, Figure EV3B-F and Appendix Figure S2-3). SRSF3 depletion had no effect on the total-ΔCNNC miR-17-92 reactivity profile and affected the 17/20a-ΔCNNC miR-17-92 SHAPE reactivities only downstream of the two CNNC sites that were not mutated, demonstrating that SRSF3 acted on miR-17-92 structure by binding to the CNNC sites (Figure 3G, Figure EV3E). *In silico* sequence based minimum free energy structure predictions suggested that the introduced point mutations altered the thermodynamical properties of miR-17-92 RNA (Appendix Figure S2A-C), complicating the direct comparisons of the WT and total-ΔCNNC secondary structures. Despite this, the effects of SRSF3 depletion and SRSF3 binding site mutations on the SHAPE reactivity ratios were remarkably similar (Figure 3J, Figure EV3F). Thus, the miR-17-92 secondary structure could be modulated either by SRSF3 depletion or SRSF3 binding site mutations. The additional SHAPE-MaP experiments support our conclusion that SRSF3 binding to the CNNC sites modulates the miR-17-92 secondary structure.

**Advisor's comments:**

I agree that it remains unclear how SRSF3 changes the pri-miRNA structure. However, as long as the SHAPE data is clear enough to indicate the rearrangement of the secondary structure, it might be OK. It would be ideal if the authors can design a mutation(s) that drives/stablizes the rearranged secondary structure (based on the SHAPE data) and show enhanced production of miR-17/20a, but I'm not sure how feasible it is.

Designing mutations that alter the secondary structure of a long RNA is challenging as both local and distal base pairing interactions need to be considered within a long RNA, increasing the number of possible RNA structures. As shown by our data, local base pairing alterations had global effects on the secondary structure. In addition, tertiary contacts (such as protein-protein interactions) that are not directly measured by SHAPE-MaP play a role in the cellular context. Our data demonstrates that cellular interactions supersede the simple thermodynamical

properties of RNA. For instance, in the *in silico* predicted minimum free energy miR-17-92 structures based on thermodynamical properties of nucleic acids alone, the miR-18a stem loop did not form correctly although it was correctly formed in all SHAPE-MaP based secondary structures (Appendix Figure 2A-C, Appendix Figure 3B-C, Figure 3B). Based on our data we propose that SRSF3 binding to CNNC sites within miR-17-92 disrupts base pairing that results in a secondary structure favouring miR-17/20a processing. Following SRSF3 depletion, the CNNC sites downstream of miR-17/20a base paired (Figure 3E), while in total-ΔCNNC mutant these regions were single-stranded because the mutated sequences were not able to base pair with the previously complementary sequences (Figure 3I). However, the effects of SRSF3 depletion and SRSF3 binding site mutations on the SHAPE reactivity ratios were remarkably similar (Figure 3J, Figure EV3F), suggesting that the altered base pairing around the CNNC sites resulted in similar global secondary structure alterations, as well as potentially changes in tertiary contacts. It will be interesting to further investigate in the future how we could rationally design mutations to control RNA secondary and tertiary structures in the cellular context to regulate gene expression. Our work provides a starting point for such investigations.

**1st Revision - Editorial Decision** **21st Apr 2023**

Dear Prof. Änkö,

Thank you for the submission of your revised manuscript. We have now received the enclosed reports from the referees. Referee 2 still has one more suggestion that I would like you to address before we can proceed with the official acceptance of your manuscript.

In line with referee 2's comments, the title of the manuscript also needs to be corrected. Please also carefully edit the abstract again to make sure that all overstatements are removed. Your new findings in the abstract need to be described in present tense, as per journal policy.

A few other editorial requests will also need to be addressed:

- Please add up to 5 keywords to the ms file.

- The conflict of interest subheading needs to be corrected to "Disclosure and Competing Interest Statement"

- The reference format needs to be corrected to Harvard/EMBO reports style.

- Fig 3D and EV3D callouts are missing, please add.

- I attach to this email a related ms file with comments by our data editors. Please address all comments in the final ms.

EMBO press papers are accompanied online by A) a short (1-2 sentences) summary of the findings and their significance, B) 2-3 bullet points highlighting key results and C) a synopsis image that is exactly 550 pixels wide and 200-600 pixels high (the height is variable). You can either show a model or key data in the synopsis image. Please note that text needs to be readable at the final size. Please send us this information along with the final manuscript.

I look forward to seeing a final version of your manuscript as soon as possible. Please use this link to submit your revision: https://embor.msubmit.net/cgi-bin/main.plex

Best regards,
Esther

Esther Schnapp, PhD
Senior Editor
EMBO reports

Referee #1:

The authors performed a thorough revision and have addressed the minor concerns and suggestions that were raised during the first round of review. Thus, I remain enthusiastic about this study. I do recommend publication in EMBO reports.

Referee #2:

This manuscript has been greatly improved with the much-needed rescue assays. However, my previous concerns are not fully addressed.

The primary shortcoming is that the proposed model (SRSF3 selectively promotes miR-17/20 via modulating the structure of their parental transcript) has not been definitively proven. While reduction of only miR-17/20 upon SRSF3 depletion is clear in the initial observation, this selective nature is not consistent in following reporter/mutagenesis studies and rescue assays. This indicates other factors also contributing to the biogenesis of this cluster. Furthermore, the Shape date does not provide a clear explanation why miR-17/20 are SELECTIVELY impacted. Finally, without mutations which can lock the "changed" structure, we cannot exclude the possibility that SRSF3 regulates miR-17/92 cluster biogenesis independent of RNA structure changes.

Authors should discuss these limitations in the manuscript.

The authors have addressed all minor editorial requests.

## 2nd Revision - Editorial Decision 15th May 2023

Prof. Minna-Liisa Änkö
Tampere University
Faculty of Medicine and Health Technology
Arvo Ylpon katu 34
Tampere, Pirkanmaa 33530
Finland

Dear Prof. Änkö,

I am very pleased to accept your manuscript for publication in the next available issue of EMBO reports. Thank you for your contribution to our journal.

At the end of this email I include important information about how to proceed. Please ensure that you take the time to read the information and complete and return the necessary forms to allow us to publish your manuscript as quickly as possible.

As part of the EMBO publication's Transparent Editorial Process, EMBO reports publishes online a Review Process File to accompany accepted manuscripts. As you are aware, this File will be published in conjunction with your paper and will include the referee reports, your point-by-point response and all pertinent correspondence relating to the manuscript.

If you do NOT want this File to be published, please inform the editorial office within 2 days, if you have not done so already, otherwise the File will be published by default [contact: emboreports@embo.org]. If you do opt out, the Review Process File link will point to the following statement: "No Review Process File is available with this article, as the authors have chosen not to make the review process public in this case."

Thank you again for your contribution to EMBO reports and congratulations on a successful publication. Please consider us again in the future for your most exciting work.

Best regards,
Esther

Esther Schnapp, PhD
Senior Editor
EMBO reports

*********************************************************************************

THINGS TO DO NOW:

Please note that you will be contacted by Wiley Author Services to complete licensing and payment information. The required 'Page Charges Authorization Form' is available here: https://www.embopress.org/pb-assets/embo-site/er_apc.pdf - please download and complete the form and return to embopressproduction@wiley.com

EMBO Press participates in many Publish and Read agreements that allow authors to publish Open Access with reduced/no publication charges. Check your eligibility: https://authorservices.wiley.com/author-resources/Journal-Authors/open-access/affiliation-policies-payments/index.html

You will receive proofs by e-mail approximately 2-3 weeks after all relevant files have been sent to our Production Office; you should return your corrections within 2 days of receiving the proofs.

Please inform us if there is likely to be any difficulty in reaching you at the above address at that time. Failure to meet our deadlines may result in a delay of publication, or publication without your corrections.

All further communications concerning your paper should quote reference number EMBOR-2022-56021V3 and be addressed to emboreports@wiley.com.

Should you be planning a Press Release on your article, please get in contact with emboreports@wiley.com as early as possible, in order to coordinate publication and release dates.

## EMBO Press Author Checklist


| | |
|---|---|
| Corresponding Author Name: Minna-Liisa Änkö | |
| Journal Submitted to: EMBO Reports | |
| Manuscript Number: EMBOR-2022-56021V1 | |


**USEFUL LINKS FOR COMPLETING THIS FORM**
The EMBO Journal - Author Guidelines
EMBO Reports - Author Guidelines
Molecular Systems Biology - Author Guidelines
EMBO Molecular Medicine - Author Guidelines

**Reporting Checklist for Life Science Articles (updated January 2022)**
This checklist is adapted from Materials Design Analysis Reporting (MDAR) Checklist for Authors. MDAR establishes a minimum set of requirements in transparent reporting in the life sciences (see Statement of Task: 10.31222/osf.io/9sm4x). Please follow the journal's guidelines in preparing your manuscript.
**Please note that a copy of this checklist will be published alongside your article.**

**Abridged guidelines for figures**
**1. Data**
The data shown in figures should satisfy the following conditions:
- the data were obtained and processed according to the field's best practice and are presented to reflect the results of the experiments in an accurate and unbiased manner.
- ideally, figure panels should include only measurements that are directly comparable to each other and obtained with the same assay.
- plots include clearly labeled error bars for independent experiments and sample sizes. Unless justified, error bars should not be shown for technical replicates.
- if n<5, the individual data points from each experiment should be plotted. Any statistical test employed should be justified.
- Source Data should be included to report the data underlying figures according to the guidelines set out in the authorship guidelines on Data Presentation.

**2. Captions**
Each figure caption should contain the following information, for each panel where they are relevant:
- a specification of the experimental system investigated (eg cell line, species name).
- the assay(s) and method(s) used to carry out the reported observations and measurements.
- an explicit mention of the biological and chemical entity(ies) that are being measured.
- an explicit mention of the biological and chemical entity(ies) that are altered/varied/perturbed in a controlled manner.
- the exact sample size (n) for each experimental group/condition, given as a number, not a range;
- a description of the sample collection allowing the reader to understand whether the samples represent technical or biological replicates (including how many animals, litters, cultures, etc.).
- a statement of how many times the experiment shown was independently replicated in the laboratory.
- definitions of statistical methods and measures:
  - common tests, such as t-test (please specify whether paired vs. unpaired), simple χ2 tests, Wilcoxon and Mann-Whitney tests, can be unambiguously identified by name only, but more complex techniques should be described in the methods section;
  - are tests one-sided or two-sided?
  - are there adjustments for multiple comparisons?
  - exact statistical test results, e.g., P values = x but not P values < x;
  - definition of 'center values' as median or average;
  - definition of error bars as s.d. or s.e.m.

**Please complete ALL of the questions below.**
**Select "Not Applicable" only when the requested information is not relevant for your study.**

**Materials**

| Newly Created Materials | Information included in the manuscript? | In which section is the information available? (Reagents and Tools Table, Materials and Methods, Figures, Data Availability Section) |
|---|---|---|
| New materials and reagents need to be available; do any restrictions apply? | Yes | Data availability section, Materials and methods, Tables |

| Antibodies | Information included in the manuscript? | In which section is the information available? (Reagents and Tools Table, Materials and Methods, Figures, Data Availability Section) |
|---|---|---|
| For **antibodies** provide the following information:<br>- Commercial antibodies: RRID (if possible) or supplier name, catalogue number and or/clone number<br>- Non-commercial: RRID or citation | Yes | Materials and methods (reagents) |

| DNA and RNA sequences | Information included in the manuscript? | In which section is the information available? (Reagents and Tools Table, Materials and Methods, Figures, Data Availability Section) |
|---|---|---|
| **Short novel DNA or RNA including primers, probes**: provide the sequences. | Yes | Materails and methods, Tables |

| Cell materials | Information included in the manuscript? | In which section is the information available? (Reagents and Tools Table, Materials and Methods, Figures, Data Availability Section) |
|---|---|---|
| **Cell lines:** Provide species information, strain. Provide accession number in repository **OR** supplier name, catalog number, clone number, and/**OR** RRID. | Yes | Materials and methods |
| **Primary cultures:** Provide species, strain, sex of origin, genetic modification status. | Not Applicable | |
| Report if the cell lines were recently **authenticated** (e.g., by STR profiling) and tested for mycoplasma contamination. | Yes | Tested for mycoplasma |

| Experimental animals | Information included in the manuscript? | In which section is the information available? (Reagents and Tools Table, Materials and Methods, Figures, Data Availability Section) |
|---|---|---|
| **Laboratory animals or Model organisms:** Provide species, strain, sex, age, genetic modification status. Provide accession number in repository **OR** supplier name, catalog number, clone number, **OR** RRID. | Not Applicable | |
| **Animal observed in or captured from the field:** Provide species, sex, and age where possible. | Not Applicable | |
| Please detail **housing and husbandry conditions**. | Not Applicable | |

| Plants and microbes | Information included in the manuscript? | In which section is the information available? (Reagents and Tools Table, Materials and Methods, Figures, Data Availability Section) |
|---|---|---|
| **Plants:** provide species and strain, ecotype and cultivar where relevant, unique accession number if available, and source (including location for collected wild specimens). | Not Applicable | |
| **Microbes:** provide species and strain, unique accession number if available, and source. | Not Applicable | |

| Human research participants | Information included in the manuscript? | In which section is the information available? (Reagents and Tools Table, Materials and Methods, Figures, Data Availability Section) |
|---|---|---|
| If collected and within the bounds of privacy constraints report on age, sex and gender or ethnicity for all study participants. | Not Applicable | |

| Core facilities | Information included in the manuscript? | In which section is the information available? (Reagents and Tools Table, Materials and Methods, Figures, Data Availability Section) |
|---|---|---|
| If your work benefited from core facilities, was their service mentioned in the acknowledgments section? | Yes | Acknowledgements section |

**Design**

| Study protocol | Information included in the manuscript? | In which section is the information available? (Reagents and Tools Table, Materials and Methods, Figures, Data Availability Section) |
|---|---|---|
| If study protocol has been **pre-registered, provide DOI in the manuscript**. For clinical trials, provide the trial registration number **OR** cite DOI. | Not Applicable | |
| Report the **clinical trial registration number** (at ClinicalTrials.gov or equivalent), where applicable. | Not Applicable | |

| Laboratory protocol | Information included in the manuscript? | In which section is the information available? (Reagents and Tools Table, Materials and Methods, Figures, Data Availability Section) |
|---|---|---|
| Provide DOI OR other citation details if **external detailed step-by-step protocols** are available. | Yes | Materials and methods |

| Experimental study design and statistics | Information included in the manuscript? | In which section is the information available? (Reagents and Tools Table, Materials and Methods, Figures, Data Availability Section) |
|---|---|---|
| Include a statement about **sample size** estimate even if no statistical methods were used. | Yes | Figure legends |
| Were any steps taken to minimize the effects of subjective bias when allocating animals/samples to treatment (e.g. **randomization procedure**)? If yes, have they been described? | Not Applicable | |
| Include a statement about **blinding** even if no blinding was done. | Not Applicable | |
| Describe **inclusion/exclusion criteria** if samples or animals were excluded from the analysis. Were the criteria pre-established?<br><br>If sample or data points were omitted from analysis, report if this was due to attrition or intentional exclusion and provide justification. | Not Applicable | |
| For every figure, are **statistical tests** justified as appropriate? Do the data meet the assumptions of the tests (e.g., normal distribution)? Describe any methods used to assess it. Is there an estimate of variation within each group of data? Is the variance similar between the groups that are being statistically compared? | Yes | The data met the assumptions of the tests. Variance is similar between compared groups. |

| Sample definition and in-laboratory replication | Information included in the manuscript? | In which section is the information available? (Reagents and Tools Table, Materials and Methods, Figures, Data Availability Section) |
|---|---|---|
| In the figure legends: state number of times the experiment was **replicated** in laboratory. | Yes | Figure legends |
| In the figure legends: define whether data describe **technical or biological replicates**. | Yes | Materials and methods |

## Ethics

| Ethics | Information included in the manuscript? | In which section is the information available? (Reagents and Tools Table, Materials and Methods, Figures, Data Availability Section) |
|---|---|---|
| Studies involving **human participants**: State details of **authority granting ethics approval** (IRB or equivalent committee(s), provide reference number for approval. | Yes | Materials and methods |
| Studies involving **human participants**: Include a statement confirming that **informed consent** was obtained from all subjects and that the experiments conformed to the principles set out in the WMA Declaration of Helsinki and the Department of Health and Human Services Belmont Report. | Yes | Materials and methods |
| Studies involving **human participants:** For publication of **patient photos**, include a statement confirming that consent to publish was obtained. | Not Applicable | |
| Studies involving experimental **animals**: State details of **authority granting ethics approva**l (IRB or equivalent committee(s), provide reference number for approval. Include a statement of compliance with ethical regulations. | Not Applicable | |
| Studies involving **specimen and field samples:** State if relevant **permits** obtained, provide details of authority approving study; if none were required, explain why. | Not Applicable | |

| Dual Use Research of Concern (DURC) | Information included in the manuscript? | In which section is the information available? (Reagents and Tools Table, Materials and Methods, Figures, Data Availability Section) |
|---|---|---|
| Could your study fall under dual use research restrictions? Please check biosecurity documents and list of **select agents and toxins** (CDC): https://www.selectagents.gov/sat/list.htm | Not Applicable | |
| If you used a select agent, is the security level of the lab appropriate and reported in the manuscript? | Not Applicable | |
| If a study is subject to dual use research of concern regulations, is the name of the **authority granting approval and reference number** for the regulatory approval provided in the manuscript? | Not Applicable | |

## Reporting

The MDAR framework recommends adoption of discipline-specific guidelines, established and endorsed through community initiatives. Journals have their own policy about requiring specific guidelines and recommendations to complement MDAR.

| Adherence to community standards | Information included in the manuscript? | In which section is the information available? (Reagents and Tools Table, Materials and Methods, Figures, Data Availability Section) |
|---|---|---|
| State if relevant guidelines or checklists (e.g., **ICMJE, MIBBI, ARRIVE, PRISMA**) have been followed or provided. | Not Applicable | |
| For **tumor marker prognostic studies**, we recommend that you follow the **REMARK** reporting guidelines (see link list at top right). See author guidelines, under 'Reporting Guidelines'. Please confirm you have followed these guidelines. | Not Applicable | |
| For **phase II and III randomized controlled trials**, please refer to the **CONSORT** flow diagram (see link list at top right) and submit the CONSORT checklist (see link list at top right) with your submission. See author guidelines, under 'Reporting Guidelines'. Please confirm you have submitted this list. | Not Applicable | |

## Data Availability

| Data availability | Information included in the manuscript? | In which section is the information available? (Reagents and Tools Table, Materials and Methods, Figures, Data Availability Section) |
|---|---|---|
| Have **primary datasets** been deposited according to the journal's guidelines (see 'Data Deposition' section) and the respective accession numbers provided in the Data Availability Section? | Yes | Data availability section |
| Were **human clinical and genomic datasets** deposited in a public access-controlled repository in accordance to ethical obligations to the patients and to the applicable consent agreement? | Not Applicable | |
| Are **computational models** that are central and integral to a study available without restrictions in a machine-readable form? Were the relevant accession numbers or links provided? | Not Applicable | |
| If publicly available data were reused, provide the respective **data citations in the reference list**. | Yes | Materials and methods, Reference list |
